# Population structure and demographic history of the gastropod *Thaisella chocolata* (Duclos, 1832) from the Southeast Pacific inferred from mitochondrial DNA analyses

**DOI:** 10.1002/ece3.9276

**Published:** 2022-09-09

**Authors:** Eliana Zelada‐Mázmela, Lorenzo E. Reyes‐Flores, Julissa J. Sánchez‐Velásquez, Claudia Ingar, Luis E. Santos‐Rojas

**Affiliations:** ^1^ Laboratory of Genetics, Physiology, and Reproduction, Faculty of Sciences Universidad Nacional del Santa Nuevo Chimbote Peru

**Keywords:** 16S rRNA, cytochrome oxidase subunit 1, genetic diversity, phylogeography, population structure, *Thaisella chocolata*

## Abstract

The present‐day population structure of a species reflects the combination of oceanographic currents, life‐history traits, and historical events. However, little is known about the mechanisms that have shaped the gene lineage distribution of marine species inhabiting the Southeast Pacific. Here, we provide a comprehensive phylogeographical study of a species distributed along the Southeast Pacific coastal region by analyzing the endemic gastropod *Thaisella chocolata* (Duclos, 1832). Sequencing of mitochondrial cytochrome c oxidase subunit 1 (*CO1*) and 16S rRNA revealed strikingly high haplotypic nucleotide and genetic diversity but a lack of significant population differentiation within the survey area. In addition, a star‐shaped phylogeny and significantly negative Tajima's D and Fu's Fs tests of neutrality suggested historical occurrence of rapid demographic expansion. Mismatch distributions and Bayesian inference analyses also confirmed *T. chocolata* to have undergone two ancestral demographic expansions. Calculations suggested that these expansions began in the lower and middle Pleistocene epoch, likely due to continental shelf development and climatic conditions. These findings could help establish a genetic baseline for *T. chocolata* as the first step toward sustainable spatial management of this species, as well as understand this species’ response to future climate change.

## Graphical abstract

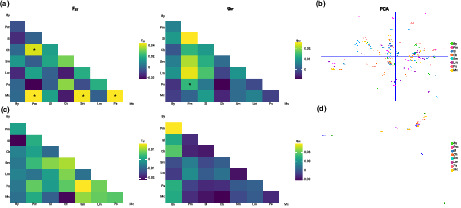

## 1 INTRODUCTION

An essential component of long‐term fisheries management is the determination of a species' genetic population structure, which commonly reflects the combination of oceanographic currents, life‐history traits, and historical events (Barahona et al., 2017). In the Southeast Pacific, climatic and geological events especially have played significant roles in modifying species assemblages and species ranges (Cárdenas, Castilla, et al., 2016; Cárdenas, Silva, et al., 2009). However, distinct from the Atlantic and the Northeast Pacific, how oceanographic factors and historical events have helped shape the region's rich biodiversity is poorly documented. One of the most important geological events in the Southeast Pacific is the appearance of the modern Humboldt Current System (HCS), one of the four major global eastern boundary currents (Hill et al., 1998; Zinsmeister, 1978). The HCS is a strong coastal upwelling ecosystem that derives from the West Wind Drift (WWD) at around 40° S and flows northwards along the Pacific eastern seaboard as part of the Coriolis‐force‐induced South Pacific gyre (Gutiérrez et al., 2016). The HCS comprises four major currents: the combination of the Peru and Chile coastal currents that exist over the shelf area and reach only to shallow depths; the Peru–Chile current found at the boundary between the upwelling and downwelling regions; the Peru–Chile Countercurrent, a poleward flow that brings cold and nutrient‐rich water to the surface from depths below 150 m; and the Peru–Chile undercurrent typically found at depths between 50 and 4400 m (Karstensen & Ulloa, 2009). By creating regional geographic barriers, these currents can influence local adaptation in marine species populations; for example, Cárdenas et al. (2016) proposed that the two spatial genetic patterns found in the marine gastropod *Concholepas concholepas* along the length of the HCS could be an effect of the WWD current, which splits at approximately 43° S into the equator‐ward Peru Current and the pole‐ward Cape Horn Current. In addition, being geographically situated near the eastern terminus of the Pacific equatorial wave guide, the HCS bears the immediate brunt of the El Niño–Southern Oscillation (ENSO), an interannual climate variability that causes strong fluctuations in species abundance and dynamics (De Oliveira et al., 2009; Martínez et al., 2003). During the warm phase of an ENSO event, the wind field changes at the equator and the warm waters of the western equatorial Pacific region propagate to the eastern Pacific by means of Kelvin waves that radiate poleward along the eastern boundary in coastal trapped waves (Philander, 1983; Thiel et al., 2007). The HCS is thereby subjected to intermittent drastic environmental rearrangements more extreme than those known to occur in any other sizeable marine ecosystem on Earth (Bakun & Weeks, 2008).

Because the HCS has been a permanent oceanographic feature in the Southeast Pacific throughout most of the Cenozoic, the geologic record along the coasts of Peru and Chile should contain a detailed history of past climatic events (Zinsmeister, 1978). In fact, the marine species inhabiting this region evidence genetic signatures that reflect demographic changes driven by geological and oceanographic changes during the Tertiary and early Quaternary (Cárdenas, Castilla, et al., 2016; Cárdenas, Silva, et al., 2009). For example, prior to the development of the West Antarctic ice sheet, the prevailing westerly winds would have been far weaker, with the net result of a southward shift of the Polar Front and a corresponding southward displacement of the WWD (Zinsmeister, 1978). This more southerly placement may have led to prevailing wind patterns along the Southeast Pacific coasts to promote the development of a strong, warm, south‐flowing, ENSO‐like countercurrent along Peru and Chile (Zinsmeister, 1978). The presence of subtropical molluscan faunas as far south as the Golfo de Penas, Chile, in the Middle and Early Miocene, and the presence of many extralimital species in the Antofagasta‐Mejillones area, Chile, during the Marine Isotope Stage 11 interglacial (MIS‐11) suggests that ENSO conditions indeed existed (Berger & Wefer, 2003; Zinsmeister, 1978). It is envisioned that during the MIS‐11 interglacial (426–396 ka before present [BP]), the climatic regime of northern Chile was characterized by significantly warmer air temperature, increased solar radiation, reduced cloud cover, and enhanced strong, quasi‐periodical, ENSO‐like conditions (Ortlieb et al., 2003; Tzedakis et al., 2022). If this hypothesis is correct, warmer conditions would have intermittently existed much farther south along South America (Zinsmeister, 1978). As mentioned above, ENSO events may become biological disasters affecting populations' genetic signatures (De Oliveira et al., 2009; Martínez et al., 2003). If a single event can have such an impact, the question arises as to what the cumulative effects may be over a longer time scale (Ibáñez et al., 2012).

Glacial and interglacial periods and consequent eustatic sea‐level oscillations during the Late Pleistocene also influenced the distribution and population sizes of temperate marine species inhabiting the Southeast Pacific (Barahona et al., 2017). The interval spanning from the Last Glacial Maximum (LGM, 21–18 ka BP) to the preindustrial era represents the most recent large‐scale reorganization of the climate system (Osman et al., 2021). During this time, an abrupt decline in sea level, to about 120–140 m below current sea level, induced a massive exposure of the continental shelf that left genetic signatures at population and species levels congruent with population decline (Lambeck et al., 2002; Salvatteci, 2013). In the Southeast Pacific, for example, analysis of laminated sediments of the Peruvian continental shelf revealed minimum abundances of oceanic species during the LGM (Salvatteci, 2013). Then, at the beginning of the Younger Dryas period (12.9–11.8 ka), during which the sea level rose, the primary productivity off Peru increased (Rein et al., 2005). These fluctuating paleoclimatic conditions can be expected to have consequences on both demography and population structure (Barahona et al., 2017; Echevin et al., 2011). For the Southeast Pacific, however, most biogeographical studies to date have focused on the description of species population structures without a deep phylogeographical assessment of the mechanisms that could have generated the observed patterns. This limits our understanding of the historical and contemporary processes that led to the present‐day distribution of the species in question. To address this issue, in this study, we selected a species with a population history that can be traced back to the latest part of the Pliocene epoch and that displays a geographical range covering the entire Peruvian coastal region, including the transition zone between the Peruvian and the Panamanian biogeographical provinces: the marine gastropod *Thaisella chocolata* (Duclos, 1832) (DeVries, 2007; Ganoza‐Chozo et al., 2021; Osorio‐Ruíz, 2002; Tarazona et al., 2003). On account of being endemic to the Southeast Pacific coast and having a life history that includes a planktonic larval stage of 4 months (Ganoza‐Chozo et al., 2021; Osorio‐Ruíz, 2002; Romero et al., 2004), *T. chocolata*, an inhabitant of the rocky intertidal zone of South America, is ideal for studying the historical and contemporary process that led to its present‐day distribution and the effects of sharp contrasts among biogeographical areas. Moreover, *T. chocolata* is a heavily exploited gastropod, with extraction records going back to the 1970s (Flores et al., 1998; Ganoza‐Chozo et al., 2021; Promperú, 2019). Therefore, identification of potential subpopulations of this species, as in this study, may provide an important regional context in which to frame marine management or conservation efforts. Conservation efforts are especially necessary since the Peruvian population of *T. chocolata* has rapidly decreased due to fishing pressure and anthropogenic disturbances (Alfaro‐Mudarra, 2020).

Even though marine populations may consist of ample numbers of individuals to start showing declines in genetic diversity measurable with feasible sample sizes (Ryman et al., 1995), the effective population size, which determines the genetic properties of a population, may be orders of magnitude smaller than the census population size, for example, millions of individuals may be equivalent to an effective population size of only hundreds or thousands (Hare et al., 2011). The notion that marine species' populations lose genetic diversity is thus possible despite their large spawning stock biomasses, *which could potentially result in reduced adaptability and productivity* (Hauser et al., 2002). Considering that the Peruvian *T. chocolata* fishery is based on stocks that have already shown a reduction in population size and that the average catch for this species in the last decade is around 28% less compared with the average record of marine fishery landings reported since 1975 (Alfaro‐Mudarra, 2020; Flores et al., 1998; PRODUCE, 2021), fishery may have already caused considerable changes in genetic diversity. Determining the population's genetic diversity is, therefore, paramount to conservation and fishery decision‐making.

Genetic tools are a powerful means of examining demographic history and population dynamics; in particular, mitochondrial DNA (mtDNA) has an effective population size of approximately one‐quarter that of nuclear markers, which allows the patterns of recent historical events to be recovered without extensive sequencing efforts (Hurst & Jiggins, 2005). Moreover, although doubly uniparental inheritance (DUI) of mtDNA occurs in some invertebrate species (Ladoukakis & Zouros, 2001; Soroka, 2020), there is currently no evidence of DUI in gastropods (Gusman et al., 2017). Thus, maternal inheritance and lack of recombination also make mtDNA an appropriate marker for tracing recent evolutionary history, including colonization events and population bottlenecks, without the confounding effects of biparental inheritance and recombination inherent in nuclear DNA (Avise, 2000; Harrison, 1989; Pakendorf & Stoneking, 2005). The use of mtDNA in the study of demographic history has been especially revealing. For example, sequencing of cytochrome c oxidase subunit 1 (*CO1*) and the *mitochondrial control region has allowed the* effects of glacial and interglacial episodes to be determined among species of global commercial importance in the Southeast Pacific region, such as *Dosidicus gigas* (Ibáñez & Poulin, 2014), *Scomber japonicus* (Barahona et al., 2017), and *Trachurus murphyi* (Cárdenas, Castilla, et al., 2016; Cárdenas, Silva, et al., 2009)*. Given a clear understanding of pelagic populations' life histories and dynamics, such analyses can yield* helpful information for understanding the species' response to future climate change. In addition, *fishery management initiatives provided with such understanding are much more likely to be able to protect aspects of biocomplexity such as spawning areas and local adaptations*.

In the present study, we present the phylogeographical pattern of the marine gastropod *T. chocolata* within its native range as determined from the analysis of two mitochondrial markers, cytochrome oxidase subunit 1 (*CO1*) and 16S rRNA. Sample collection covered most of the present‐day distributional range of the species, with populations representing each of the three biogeographical regions described for the Peruvian coast: northern, central, and southern. Using population genetic analyses, we first utilized haplotype network computations and molecular‐based methods to determine the gene lineage distribution of *T. chocolata* along its range. Second, we aimed to decipher the biogeographic pathways that *T. chocolata* experienced, considering the geological and climatic history of the region. To achieve these aims, our study combined coalescent theory, multivariate analyses, Bayesian clustering, and Bayesian skyline reconstruction to facilitate a more realistic understanding of this gastropod's demographic history.

## 2 MATERIALS AND METHODS

### 2.1 Sampling, DNA extraction, and genotyping

**FIGURE 1:**
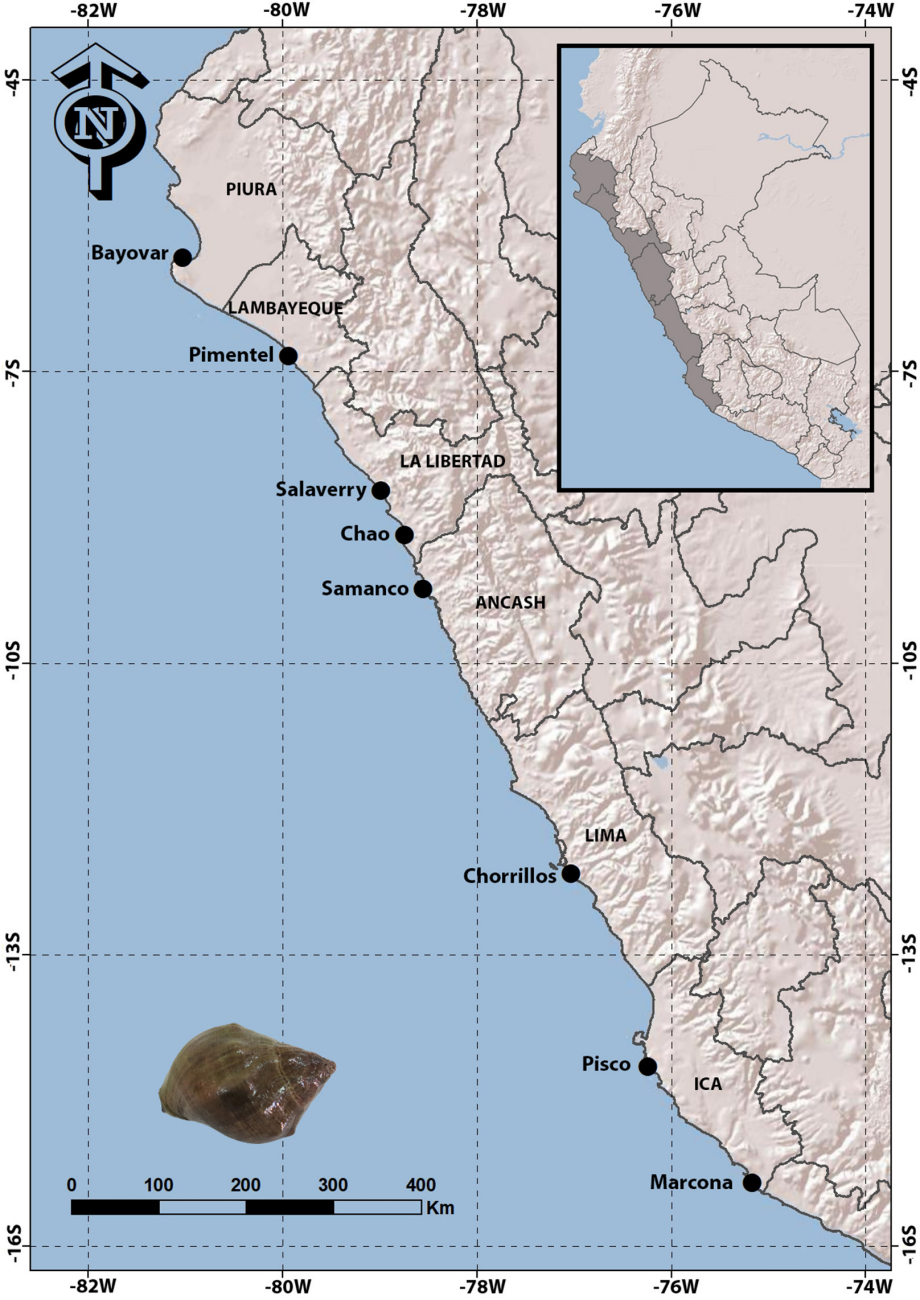
Map showing the eight locations in Peru at which *Thaisella chocolata* was sampled.

A total of 156 adult individuals of *T. chocolata* were collected from eight different locations within the northern, central, and southern regions of the Peruvian coast between 2018 and 2020 (Figure 1). Per‐location, sample sizes ranged from 18 to 22. For each captured snail, a tissue sample was collected from the foot muscle. Total genomic DNA was extracted from samples by means of proteinase K digestion followed by cetyltrimethylammonium bromide (CTAB) extraction according to a protocol adapted from Zuccarello and Lokhorst (2005). DNA quality and quantity were checked using the Epoch microplate spectrophotometer (BioTek Instruments); afterward, the concentration was adjusted to 50 ng μl^−1^. Partial regions of the mitochondrial *CO1* gene and 16S rRNA were amplified using universal primers, HCO2198 and LCO1490 for *CO1* (Folmer et al., 1994) and 16Sar and 16Sbr for 16S rRNA (Palumbi, 2005). Each amplification was performed as a 10 μl reaction, with each reaction consisting of 1 μl of *Taq* Buffer KCl‐MgCl_2_ (10×), 0.76 μl MgCl_2_ (25 mM), 0.50 μl dNTPs (2.5 mM), 0.10 μl each primer (25 μM), 6.44 μl PCR‐grade water, 0.10 μl Maximo *Taq* DNA polymerase (5 U μl^−1^) (GeneON), and 1 μl DNA template (50 ng μl^−1^). PCRs were carried out in a Veriti™ thermal cycler (Thermo Fisher Scientific). For *CO1*, PCR conditions consisted of 3‐min initial denaturation at 95°C; 35 cycles of 50 s at 95°C, 30 s at 55°C, and 1 min at 72°C; and a final extension of 7 min at 72°C. For 16S rRNA, PCR conditions consisted of 3‐min initial denaturation at 94°C; 30 cycles of 45 s at 94°C, 45 s at 55°C, and 1 min at 72°C; and a final extension of 7 min at 72°C.

Amplification success was determined by electrophoresis in 1% agarose gels. Successfully amplified products were cleaned using ExoSAP‐IT™ (Thermo Fisher Scientific), then sequenced with a BigDye™ Terminator v3.1 Cycle Sequencing Kit (Thermo Fisher Scientific) on a 3500 Genetic Analyzer (Applied Biosystems) at the sequencing facility of the Genetics, Physiology and Reproduction Laboratory of the Universidad Nacional del Santa, Chimbote, Peru. All novel *CO1* and 16S rRNA sequences generated in this study have been submitted to the GenBank database (www.ncbi.nlm.nih.gov/genbank) under accession numbers OK087138‐OK087293 and OK094932‐OK095087 (Tables S1 and S2).

### 2.2 Genetic diversity and population structure

The nucleotide sequences obtained for *T. chocolata* mitochondrial *CO1* and 16S rRNA were aligned in MAFFT 7 (Katoh & Standley, 2013) using default settings and trimmed to the shortest sequence. The number of haplotypes (*N*_*H*_), haplotype diversity (*h*), number of segregating sites (*S*), and nucleotide diversity (*π*) among samples were calculated using DnaSP 6.12.03 (Rozas et al., 2017). The number of private haplotypes unique to each population (*N*_*P*_) was determined according to the haplotype list for each population generated in Arlequin 3.5.2.2 (Excoffier & Lischer, 2010). Haplotype networks were constructed with the TCS statistical parsimony algorithm implemented in PopART 1.7 (Leigh & Bryant, 2015). To assess population structure, we estimated Wright's F‐statistic (*F*_ST_) (Weir & Cockerham, 1984) using Arlequin with statistical significance determined on the basis of 10,000 permutations. PhiST ($\Phi_{\mathrm{ST}}$), an estimator of genetic differentiation independent of mutation rate (Kronholm et al., 2010), was also computed using the R package *diveRsity* (Keenan et al., 2013), and statistical significance examined using 10,000 bootstrap iterations. Hierarchical genetic population structure was tested for with the analysis of molecular variation (AMOVA) implemented in Arlequin. To further evaluate and visualize that population genetic structure with respect to geography, principal coordinates analysis (PCoA) was conducted using DARwin 6.0.21 (Pirrier & Jacquemoud, 2006).

### 2.3 Phylogenetic analyses

Bayesian topologies were inferred using MrBayes 3.2.6 (Fredrik et al., 2012). Selection of the best nucleotide substitution model was based on the Bayesian information criterion (BIC) using jModelTest 2 (Darriba et al., 2012). For the *CO1* dataset, the chosen model utilized Hasegawa–Kishino–Yano (HKY) substitution with the gamma distribution (HKY + G). Two independent runs were performed with four Metropolis‐coupled Markov chain Monte Carlo (MCMC) chains (three hot and one cold) for 100 million generations, in which trees were sampled every 100,000 generations. Meanwhile, the chosen model for the 16S rRNA dataset was the HKY substitution model with invariable positions (HKY + I). Two independent runs were performed with four MCMC chains for 100 million generations, again with trees sampled every 100,000 generations. Convergence of MCMC chains was assessed by visual examination of the log trace of each posterior distribution, looking for a caterpillar shape and effective sample size (ESS) greater than 200 in Tracer 1.7.2 (Rambaut et al., 2014). To calculate the potential scale reduction factor and posterior probabilities, we established a burn‐in value and discarded the first 25% of trees. In both *CO1* and 16S rRNA analyses, three additional sequences of *Stramonita visceralis*, a species within the Muricidae family, were included to root the tree.

### 2.4 Haplotype accumulation curves

A nonparametric sample size estimate was determined using the R package *spider* (Brown et al., 2012). We employed random permutation subsampling via the haploAccum() function and by using the Chao 1 estimator (Chao, 1984) with the chaoHaplo() function. The latter estimates the minimum sample size capable of accounting for all haplotype diversity in a genomic dataset based on both the number of haplotypes present and the numbers of singleton and doubleton sequences (i.e., those occurring once and those appearing twice) (Brown et al., 2012; Robalo et al., 2020).

### 2.5 Population demographic history

The population demographic history and evolutionary neutrality of *T. chocolata* were assessed using two tests, Fu's Fs (Fu, 1997) and Tajima's D (Tajima, 1989), both implemented in Arlequin. *p*‐values were calculated by means of 10,000 coalescent‐based simulations. A mismatch distribution analysis using a sudden expansion model in DnaSP was conducted to compare the distribution of pairwise differences between haplotypes (Harpending, 1994; Rogers & Harpending, 1992). A Bayesian skyline plot (BSP) was also employed to examine historical demographic fluctuations since the time of the most recent common ancestor; this plot was created using Beast 3.6.4 (Suchard et al., 2018), hosted on the CIPRES Science Gateway (Miller et al., 2010). For both regions of interest, the BSP was built using a coalescent Bayesian skyline model under an HKY substitution model with empirical base frequencies and a strict molecular clock; however, certain parameters differed. For *CO1*, the substitution rate was fixed to a mutation rate of 1.57% My^−1^ previously applied in evolutionary studies of invertebrates (Wilke et al., 2009), and in the coupled MCMC analysis, the delta temperature was set to 0.1. The analysis was run in triplicate for 700 million generations with a sampling frequency of 70,000 and burn‐in of 10%. For 16S rRNA, the substitution rate was fixed to 1.4% My^−1^, a value obtained from the combined *CO1* and *16S rRNA* data (Morrison et al., 2004). The analysis was run in triplicate for 500 million generations with a sampling frequency of 50,000 and burn‐in of 10%. In both cases, the triplicate analyses were combined using LogCombiner (Suchard et al., 2018). Convergence of MCMC chains was assessed by visual examination in Tracer (Rambaut et al., 2014) of the log trace of each posterior distribution showing an ESS greater than 200. Files from all BSP analyses were collapsed into graphs using Tracer and R (R Core Team, 2020).

## 3 RESULTS

### 3.1 Population genetic analyses

The mitochondrial gene *CO1* (508 bp long) was amplified and sequenced from 156 individuals of *T. chocolata* collected along the northern, central, and southern regions of the Peruvian coast. Across the total sample, the number of haplotypes was 129, while the number of polymorphic sites was 107, of which 37 were singleton variable sites and 70 parsimoniously informative sites. This remarkable mtDNA diversity was further reflected by a haplotype diversity value close to its maximum of 1, which indicates that in the overall population, the probability of two individuals from the same locality sharing the same haplotype is <0.001%. Population‐wise, haplotype and nucleotide diversities were also very high (*h* = 0.963 ± 0.033 to 1.000 ± 0.019; *π* = 0.013 ± 0.001 to 0.016 ± 0.001) (Table 1). Even though almost every haplotype was a singleton, pairwise comparisons revealed that one of the haplotypes (Hap8) was present throughout the sampling time. Mitochondrial 16S rRNA (410 bp long) was also successfully amplified and sequenced from 155 individuals of *T. chocolata*. The number of haplotypes detected across the sample was 33, while the number of polymorphic sites was 38, of which 28 were singleton variable sites and ten parsimoniously informative sites. All populations shared at least one haplotype, and the most common haplotype (By17) was present throughout the sampling time. Population‐wise, haplotype and nucleotide diversities for this *locus* ranged from 0.284 ± 0.128 to 0.763 ± 0.103 and from 0.0007 ± 0.0004 to 0.0039 ± 0.0009, respectively (Table 1).

**TABLE 1:** Summary statistics for mitochondrial markers cytochrome c oxidase subunit 1 (*CO1*) and 16S rRNA in *Thaisella chocolata*

Population	*CO1*						16S rRNA					
	*N*	*N*_*H*_	*N*_*P*_	*h ±* SEM	*S*	*π ±* SEM	*N*	*N*_*H*_	*N*_*P*_	*h ±* SEM	*S*	*π ±* SEM
Bayovar	20	19	15	0.995 ± 0.018	36	0.0149 ± 0.0012	20	11	7	0.763 ± 0.103	16	0.0039 ± 0.0009
Pimentel	20	16	13	0.963 ± 0.033	42	0.0151 ± 0.0017	20	4	2	0.284 ± 0.128	3	0.0007 ± 0.0004
Salaverry	18	15	12	0.980 ± 0.024	39	0.0160 ± 0.0015	18	5	4	0.405 ± 0.143	6	0.0016 ± 0.0008
Chao	20	20	15	1.000 ± 0.016	34	0.0133 ± 0.0011	20	5	3	0.368 ± 0.135	5	0.0012 ± 0.0005
Samanco	19	18	18	0.994 ± 0.019	43	0.0157 ± 0.0012	18	6	2	0.562 ± 0.134	5	0.0016 ± 0.0005
Lima	19	17	15	0.988 ± 0.021	44	0.0162 ± 0.0013	19	7	6	0.608 ± 0.127	7	0.0020 ± 0.0006
Pisco	22	22	16	1.000 ± 0.014	45	0.0164 ± 0.0011	22	5	3	0.407 ± 0.128	6	0.0015 ± 0.0006
Marcona	18	18	15	1.000 ± 0.019	35	0.0127 ± 0.0014	18	5	3	0.405 ± 0.143	5	0.0014 ± 0.0006
All populations	156	129	119	0.996 ± 0.002	107	0.0152 ± 0.0005	155	33	30	0.478 ± 0.051	38	0.0018 ± 0.0002

Abbreviations: *π*, nucleotide diversity; *h*, haplotype diversity; *N*, number of samples; *N*_*H*_, number of haplotypes; *N*_*P*_, number of private haplotypes; *S*, number of segregating sites; SEM, standard error of the mean.

### 3.2 Population structure and phylogenetic analyses

**FIGURE 2:**
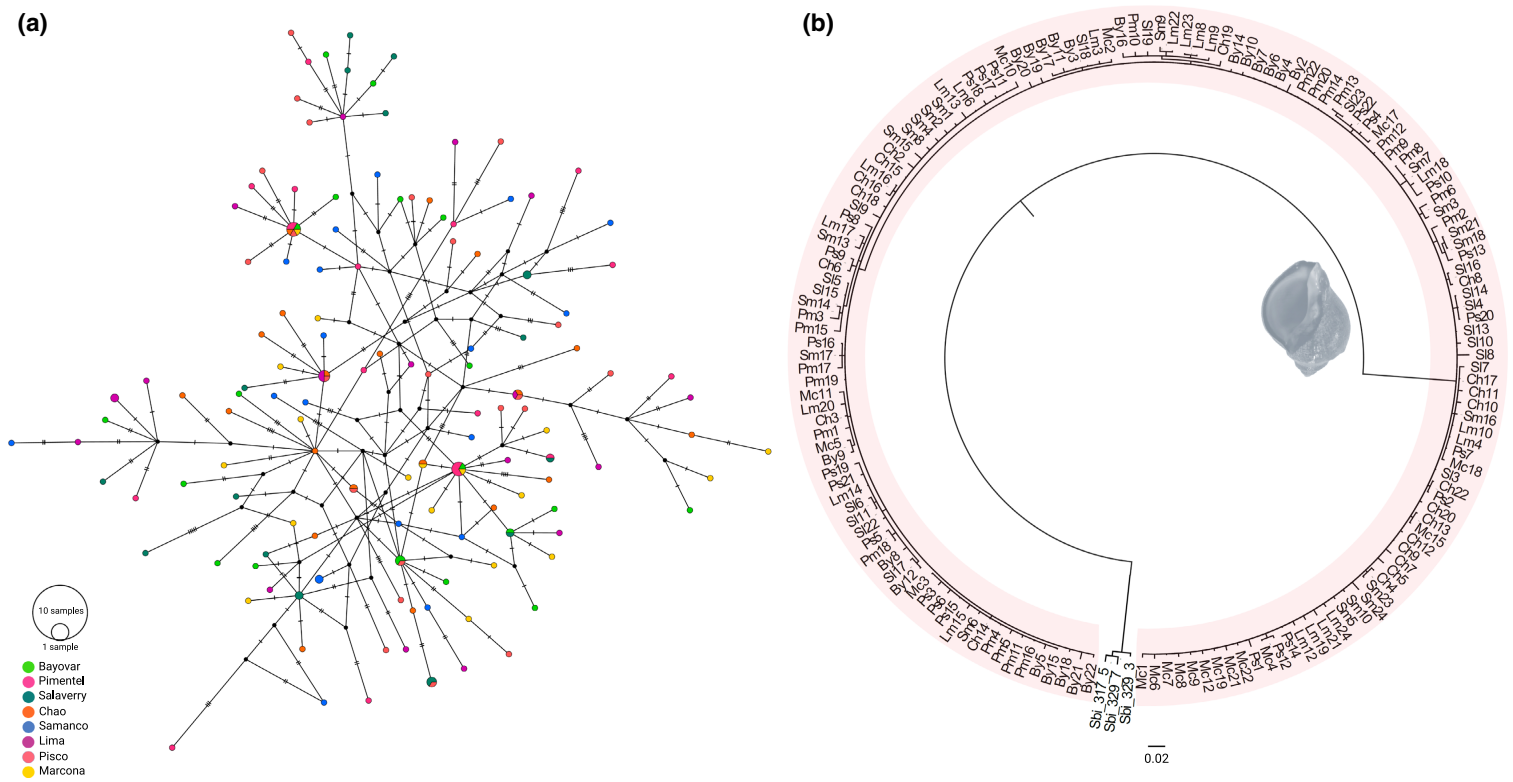
Haplotype network and phylogenetic tree based on the cytochrome c oxidase subunit 1 (*CO1*) genomic dataset. (a) The unrooted TCS haplotype network was constructed using 129 *CO1* haplotypes from *Thaisella chocolata*. Each line between points represents a single mutational step. Circle size indicates haplotype frequency. TCS network was generated using PopART. (b) Phylogenetic tree reconstructed using 156 *CO1* sequences. Bayesian inference analysis was performed for 100 million generations using MrBayes. *Stramonita biserialis* was used as an outgroup.

**FIGURE 3:**
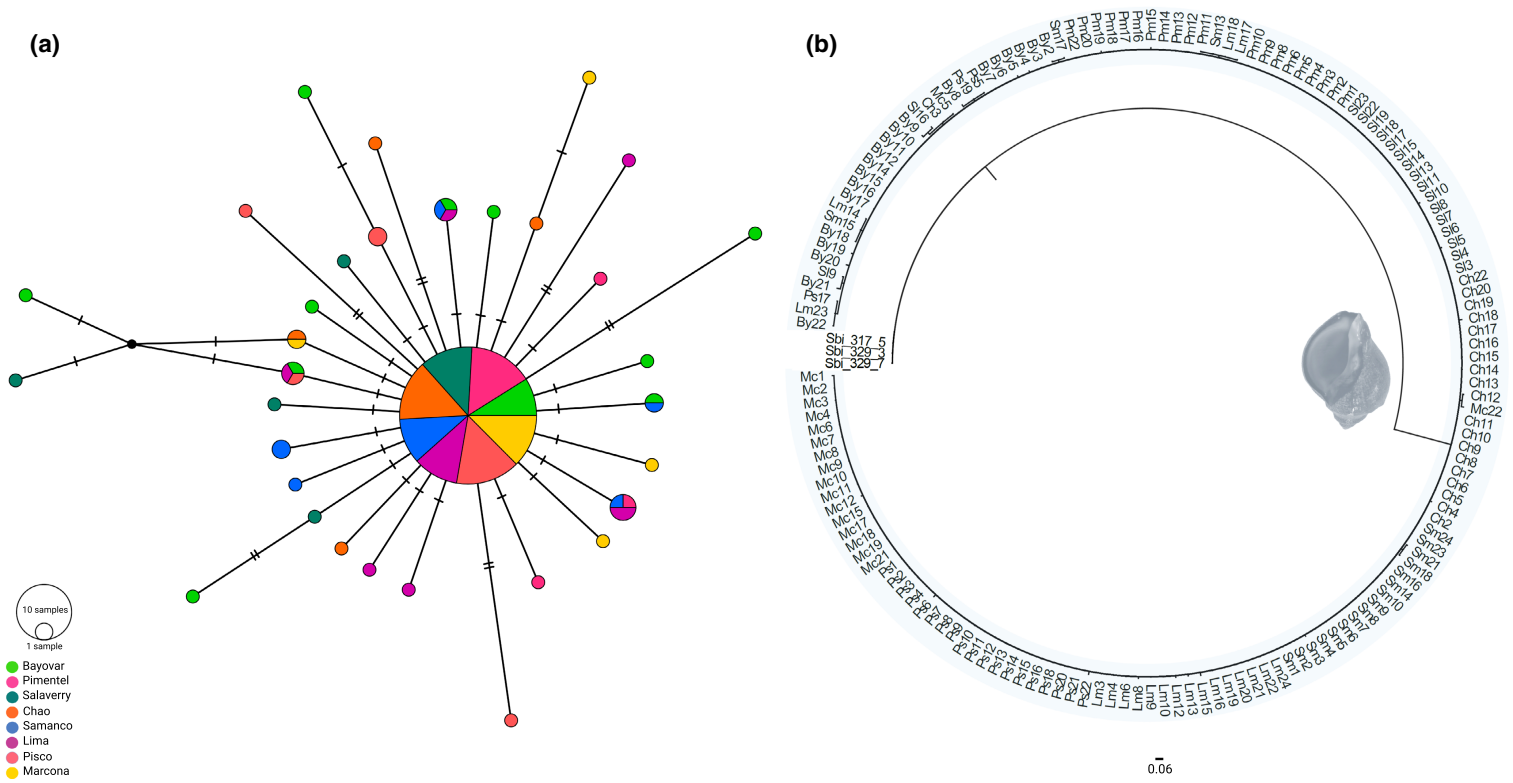
Haplotype network and phylogenetic tree based on the 16S rRNA genomic dataset. (a) The unrooted TCS haplotype network was constructed using 33 16S rRNA haplotypes from *Thaisella chocolata*. Each line between points represents a single mutational step. Circle size indicates haplotype frequency. TCS network was generated using PopART. (b) Phylogenetic tree reconstructed using 155 16S rRNA sequences. Bayesian inference analysis was performed for 100 million generations using MrBayes. *Stramonita biserialis* was used as an outgroup.

We next investigated whether population genetic structure through time and space could contribute to the mtDNA hyperdiversity observed in *T. chocolata*. At first glance, the high number of private haplotypes (Table 1) suggested Peruvian populations of this species to be strongly differentiated. By contrast, the bush‐like *CO1* network topology (Figure 2) indicated complete population mixing (Figure 2). Specifically, the topology reflects an overwhelming number of unique, private haplotypes represented by single individuals (singletons), a lack of substantive haplotype sharing between sites, and several homoplastic character states (cycles). Likewise, the haplotype network derived from 16S rRNA sequences indicated a lack of population structure, exhibiting a single star‐like pattern with dominant haplotypes at the center and low‐frequency and private haplotypes comprising the rays (Figure 3). Meanwhile, the phylogenetic trees inferred from *CO1* and 16S rRNA genomic datasets using Bayesian inference (BI) methods were unresolved. Most nodes received low bootstrap support (<50%), suggesting that for all populations examined, the sampled haplotypes lacked phylogeographic structure (Figures 2b and 3b).

**FIGURE 4:**
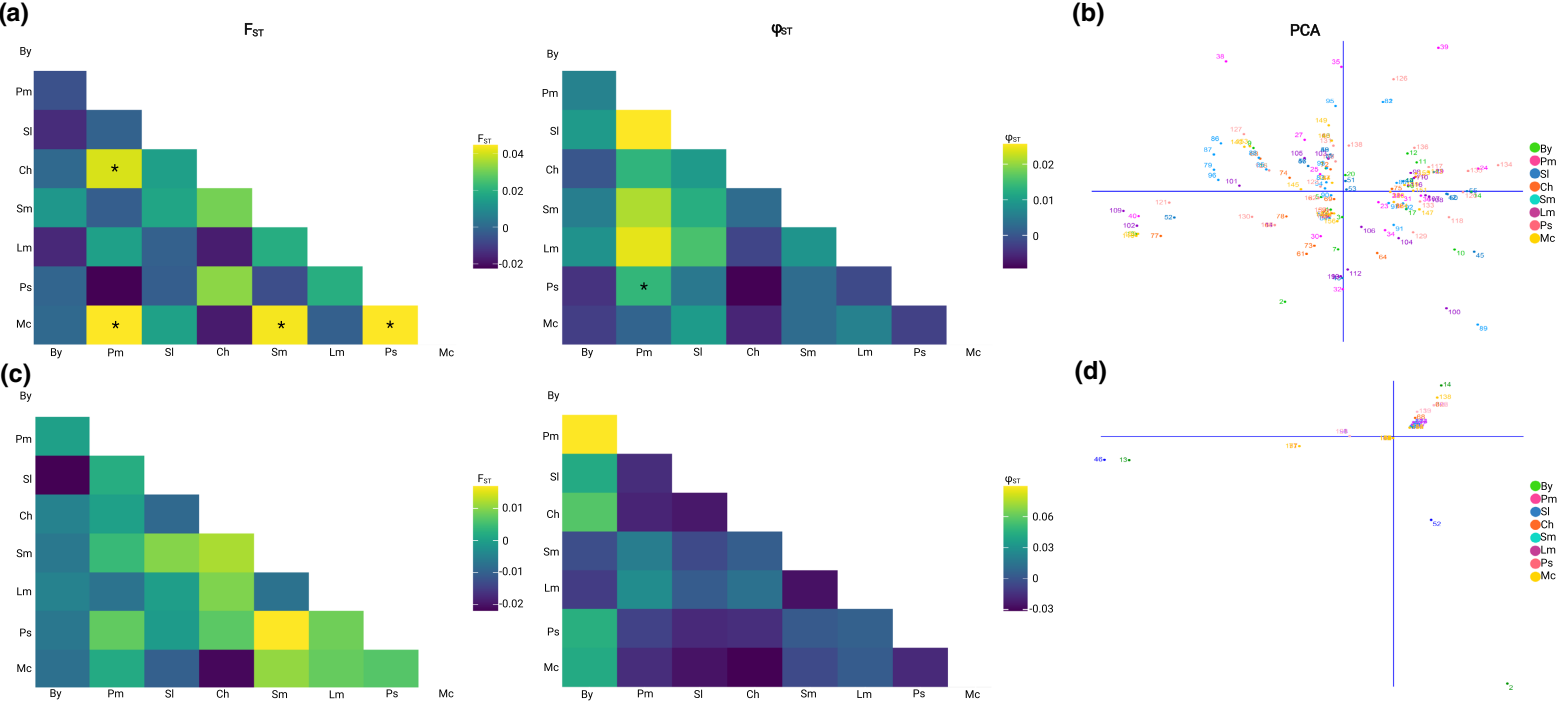
Genetic differentiation in the Peruvian population of *Thaisella chocolata*. (a) Clustered heatmaps showing *F*_ST_ and PhiST (Φ_ST_) pairwise genetic differentiation values based on cytochrome c oxidase subunit 1 (*CO1*) haplotypes from eight *T. chocolata* populations. (b) Principal coordinate analysis (PCoA) on the *CO1* dataset revealed an absence of visually discernible patterns of genetic structure. (c) Clustered heatmaps showing *F*_ST_ and Φ_ST_ pairwise genetic differentiation values based on 16S rRNA haplotypes from eight *T. chocolata* populations. (d) PCoA on the 16S rRNA dataset showed an absence of discernible patterns of genetic structure. Asterisks mark *F*_ST_ and Φ_ST_ estimates significantly greater than zero (*p* < .05). *F*_ST_ values were obtained using Arlequin. Φ_ST_ values were obtained with the R package *diveRsity*. PCoA were conducted using DARwin. By, Bayovar; pm, Pimentel; Sl, Salaverry; Ch, Chao; Sm, Samanco; Lm, Lima; Ps, Pisco; mc, Marcona.

Such an absence of population structure was further confirmed by the low values obtained for the estimates of fixation *F*_ST_ and $\Phi_{\mathrm{ST}}$ (Figure 4). Although the *CO1* genomic dataset yielded some *F*_ST_ values that differed significantly from zero (*p* > .05), those values were small, and with the low robustness of the *CO1* and 16S rRNA tree topologies, we considered the results to indicate weak genetic integrity among populations. Likewise, applying PCoA to the *CO1* and 16S rRNA genomic datasets did not reveal any visually discernible patterns (Figure 4b,d). Instead, all populations appeared to almost completely overlap with each other. Finally, evaluation of the population structure of the native range collections via AMOVA confirmed an absence of genetic structure between populations; differences between areas inferred from the *CO1* and 16S rRNA genomic datasets accounted for 0.86% y 0% of the total variance, while 99.14% and 100% was attributable to differences among individuals within each location (Table 2). These results indicate panmixia of *T. chocolata* across the geographical study area.

**TABLE 2:** Hierarchical analysis of molecular variance (AMOVA) for the cytochrome c oxidase subunit 1 (*CO1*) and 16S rRNA genomic datasets

Source of variation	*CO1*				16S rRNA			
	Df	Sum of squares	Variance components	Percentage of variation	Df	Sum of squares	Variance components	Percentage of variation
Among populations	7	31.34	0.03322	0.86	7	2.455	−0.00044	−0.12
Within populations	148	566.782	3.82961	99.14	147	52.809	0.35925	100.12
Total	155	598.122	3.86283	100	154	55.264	0.35881	100

Abbreviation: df, degrees of freedom.

### 3.3 Haplotype accumulation curves

**FIGURE 5:**
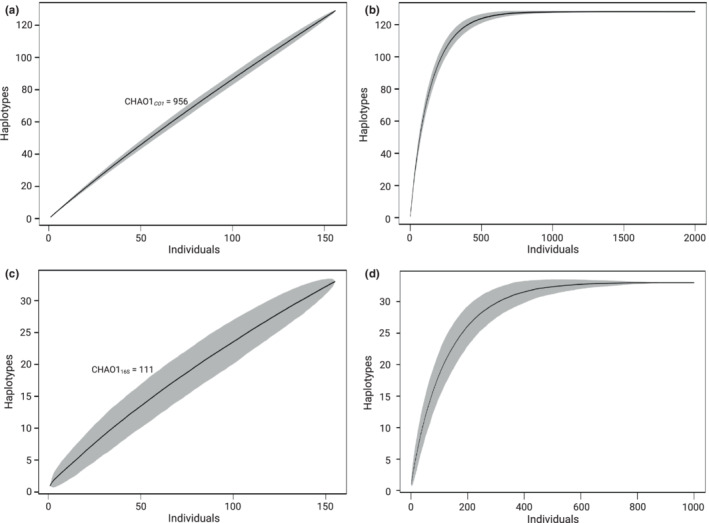
Haplotype rarefaction curves for the Peruvian population of *Thaisella chocolata*. (a) Haplotype rarefaction curve for the cytochrome c oxidase subunit 1 (*CO1*) genomic dataset, obtained using 156 sequences. (b) *CO1* haplotype rarefaction curve estimated with sample size increased to 2000 individuals. The asymptote is reached at about 900 individuals. (c) Haplotype rarefaction curve for the 16S rRNA genomic dataset, obtained using 155 sequences. (d) 16S rRNA haplotype rarefaction curve estimated with sample size increased to 1000 individuals. The asymptote is reached at about 700 individuals. Shaded areas indicate 95% confidence intervals from 1000 permutations. Figure generated using the haploAccum() and chaoHaplo() functions implemented in the R package *spider*.

**FIGURE 6:**
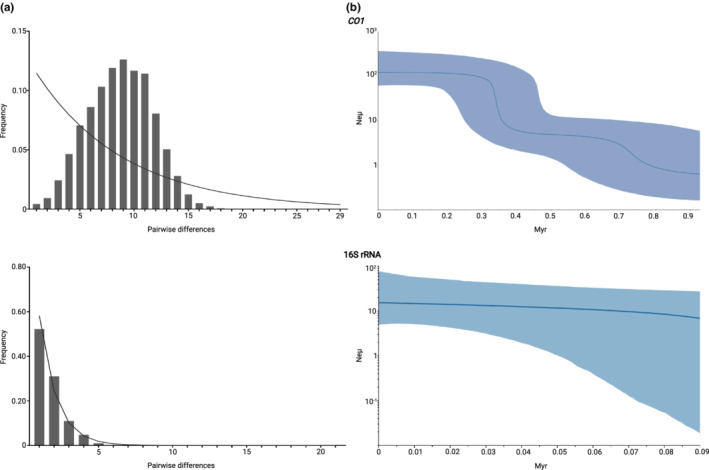
Historical demographic analyses for the Peruvian population of *Thaisella chocolata*. (a) Mismatch distribution analysis (MDA) for the cytochrome c oxidase subunit 1 (*CO1*) dataset. The MDA shows a unimodal distribution generally associated with a sudden population expansion. On the right, Bayesian skyline plot (BSP) based on the *CO1* dataset showing two waves of population expansion. (b) MDA for the 16S rRNA dataset. The MDA shows a skewed distribution associated with recent sudden expansion. On the right, BSP based on the 16S rRNA dataset showing one wave of population expansion. In MDAs, bars indicate the frequency of observed pairwise nucleotide differences, and lines the expected distribution fitted to the data under a constant population size model. In BSPs, population size is measured as the product of effective population size (ne) and mutation rate (μ). Time is expressed in million years before present (my). Solid lines indicate the median value for the relative effective population size, and solid areas denote the 95% highest posterior density intervals.

The haplotype accumulation curves for both *CO1* and 16S rRNA genomic datasets failed to reach the asymptote (Figure 5a,c), indicating that only part of the actual genetic diversity was captured. Estimates of the sample sizes needed to capture the total estimated haplotype diversity in the Peruvian population of *T. chocolata* were calculated using Chao 1; for *CO1*, the number of singletons present was determined to elevate the needed sampling to almost 900 individuals (Figure 6b), while the 16S rRNA was estimated to have a total diversity of 111 haplotypes (Figure 5c), which could be captured with a sample size of about 700 individuals (Figure 5d).

### 3.4 Demographic analysis

Fu's Fs and Tajima's D neutrality tests were performed for the *CO1* and 16S rRNA haplotypes (Table 3). In the *CO1* dataset, all populations showed significant negative values of Fu's Fs but not of Tajima's D. Meanwhile, in the 16S rRNA dataset, both measures were uniformly significantly negative. Mismatch distribution analyses revealed the *T. chocolata* population to exhibit unimodal and skewed unimodal distributions (Figure 6a). Under a neutral model, these results indicate that *T. chocolata* experienced a demographic expansion event.

**TABLE 3:** Neutrality tests for *Thaisella chocolata* based on the cytochrome c oxidase subunit 1 (*CO1*) and 16S rRNA genomic datasets

Gene	Population	Tajima's D	*p*‐value	Fu's fs	*p*‐value
*CO1*	Bayovar	−0.99876	.168	−11.69623	0
	Pimentel	−1.40492	.078	−5.416070	.017
	Salaverry	−1.14572	.120	−4.935100	.032
	Chao	−1.17319	.111	−16.15685	0
	Samanco	−1.42174	.064	−10.14247	0
	Lima	−1.39260	.075	−7.549950	.003
	Pisco	−1.27754	.095	−16.54236	0
	Marcona	−1.47807	.063	−13.88543	0
16S rRNA	Bayovar	−2.39814	.001	−7.47136	0
	Pimentel	−1.72331	.019	−2.74926	0
	Salaverry	−2.03420	.007	−2.32519	.013
	Chao	−1.97429	.005	−2.99084	0
	Samanco	−1.74211	.022	−3.85095	0
	Lima	−1.95403	.009	−4.39952	0
	Pisco	−1.91712	.010	−2.22498	.011
	Marcona	−1.95558	.008	−2.83419	.004

Patterns of fluctuation in the effective population size (*N*_*e*_) of the Peruvian population of *T. chocolata* were then examined using BSP analyses. When applied to the *CO1* dataset, these analyses revealed waves of demographic expansion to have occurred over time (Figure 6b), with a first having taken place 700,000–800,000 years before present (BP) and a second from 400,000 years BP to around 300,000 years BP. However, analysis of the 16S rRNA dataset showed only one wave of population expansion, which seemed to occur at around 90,000 years BP.

## 4 DISCUSSION

Fishery management to date has largely been concerned with the immediate resource of interest—the abundance and size of the fish available for harvesting (Allendorf et al., 1987). Little attention has been directed toward understanding the genetics of their populations, despite such understanding being crucial for determining the patterns of genetic connectivity that ultimately define the geographic structure of a species (Allendorf et al., 1987; Thiel et al., 2007). In the present study, the marine snail *T. chocolata* was found to exhibit remarkably high haplotype and nucleotide genetic diversity throughout its Peruvian geographical range, without significant population differentiation. The diversity levels were similar to values observed in other gastropod species inhabiting the HCS, such as *Concholepas concholepas* (Cárdenas, Castilla, et al., 2016; Cárdenas, Silva, et al., 2009), *Echinolittorina paytensis* (Barahona, 2017), *Siphonaria lessonii* (Fernández‐Iriarte et al., 2020; Pardo‐Gandarillas et al., 2018), and *Crepipatella fecunda* (Guzmán et al., 2011). The observed high genetic diversity of *T. chocolata* may have several explanations, of which the most plausible involve the mutation rate of mtDNA and the non‐neutral evolution hypothesis. Animal mitochondria have been shown to be inefficient, if not outright lacking, in repairing various types of DNA damage, a factor that by itself could contribute significantly to the high mutation rate observed for mtDNA (Brown et al., 1979; Clayton et al., 1974; Palit & Ngili, 2018). Thomas et al. (2010) also showed that in invertebrate eumetazoan species with shorter generation times, protein‐coding mitochondrial genes exhibit faster rates of molecular evolution as represented by nonsynonymous substitutions and fourfold degenerate transversions. This fast rate of molecular evolution could be the reason why even though positive selection has been shown to maintain low genetic diversity in mtDNA in both vertebrates and invertebrates (Bazin et al., 2006), the pattern of low genetic diversity in mtDNA does not apply to all species. Indeed, mtDNA hyperdiversity has been reported in many temperate gastropod species and highly diverse planktonic‐dispersing marine invertebrates (Fourdrilis et al., 2016). In the marine gastropod *Melarhaphe neritoides*, especially, Fourdrilis et al. (2016) reported the gene *CO1* to have a mutation rate of as high as 1.99 × 10^−4^ per site per generation, a value 1000‐ to 10,000‐fold higher than commonly estimated for mtDNA in metazoans from other phyla. This suggests that mtDNA hyperdiversity may be more common across eukaryotes than currently known. Thus, the high mutation rate of mtDNA could be the main factor driving the observed *CO1* hyperdiversity in *T. chocolata*. It is likely, however, that the rate of variation in mtDNA evolution is attributable to a more complex combination of factors than a high mutation rate alone. A second hypothesis suggests that the observed mitochondrial hyperdiversity could be explained by adaptive evolution. By evaluating the ratio of nonsynonymous to synonymous changes within and between species, Bazin et al. (2006) determined invertebrate mtDNA *loci* to exhibit a significant shift toward values less than one, consistent with adaptive evolution. In *M. neritoides*, Fourdrilis et al. (2018) found that *CO1* is under purifying selection and that among mitochondrial genes, it is most strongly subject to selection. Given this non‐neutrality, mtDNA diversity would, in many instances, reflect the time since the last occurrence of a selective sweep rather than population history and demography (Bazin et al., 2006). We should mention, however, that Quesada et al. (1998) pointed out that an excess of replacement substitutions could be explained under the nearly neutral model. In a nearly neutral scenario, the high level of purifying selection operating on mtDNA means that repeated founder events or bottlenecks associated with glacial fluctuations could lead to a striking relaxation of selective constraints on slightly deleterious mutations. Consequently, deviations from neutrality might have more to do with the timescale of selection than with a difference in the nature of evolutionary forces. Nonetheless, as Quesada et al. (1998) explained, it is difficult to exclude the hypothesis of a recent relaxation of selection on mtDNA having occurred through the reduction of associated selection coefficients, which would result in deleterious mutations becoming neutral and remaining within mtDNA lineages as polymorphisms.

Regarding genetic structure, each haplotype network constructed here displayed a large number of singletons and very few shared haplotypes among sampling sites, suggesting population differentiation. However, no associated geographical structure could be inferred from the *F*_ST_ values. It is important to note that for *CO1*, estimates of genetic differentiation based on the *F*_ST_ statistic suffer from the dataset being hypervariable. With highly variable *loci*, *F*_ST_ is restricted to values close to zero since it is determined by the amount of within‐population diversity (Kronholm et al., 2010; Meirmans & Hedrick, 2011). Nevertheless, a lack of genetic differentiation among populations was confirmed by both AMOVA and $\Phi_{\mathrm{ST}}$, an estimator independent of mutation rate (Kronholm et al., 2010). Genetic homogeneity such as that observed here in *T. chocolata* has also been shown for other marine invertebrates inhabiting the HCS with similar planktonic larval dispersal and high mtDNA variability. For example, *Echinolittorina paytensis*, a gastropod from the Littorinidae family, and *Brachidontes adamsianus*, a bivalve from the Mytilidae family, show genetic homogeneity across the Peruvian coastline (Barahona, 2017); while *Nacella magellanica*, a gastropod from the Nacellidae family, and *Cancer setosus* and *Pleuroncodes monodon*, crustaceans from the Cancridae and Munididae families, do so across the Chilean coastline (Gomez‐Uchida et al., 2003; González‐Wevar et al., 2012; Haye et al., 2010), as do *Concholepas concholepas* and *Siphonaria lessonii*, gastropods from the Muricidae and Siphonariidae families, and *Dosidicus gigas*, a cephalopod from the Ommastrephidae family, across the entire Peruvian‐Chilean coastline (Cárdenas, Castilla, et al., 2016; Cárdenas, Silva, et al., 2009; Fernández‐Iriarte et al., 2020; Ibáñez et al., 2011; Ibáñez & Poulin, 2014). The absence of geographic genetic structure observed in these species seems to obey the apparent lack of major oceanographic barriers within this region. It is also feasible that the combination of northward (Peru current) and southward (Peru–Chile countercurrent) oceanographic flows allow effective population mixing. In addition, oceanographic features of the HCS, such as the ENSO, could also contribute to genetic homogeneity (Barahona et al., 2017). During the El Niño warm phase of the ENSO pattern, warm water masses moving southward cause the transport of larvae to the south, whereas, during the cool La Niña phase, retreat of surface equatorial waters toward the north causes larval transport northward (Días & Ortlieb, 1993; Paredes et al., 1998). Together, the constant northward transport of the Humboldt current and semi‐regular southward transport mediated by the ENSO would create a bidirectional gene flow that prevents any degree of genetic differentiation for most species inhabiting the Peruvian coast (Barahona, 2017).

The genetic homogeneity observed in *T. chocolata* can also be rooted in its high potential for dispersal. The species has an intracapsular development of 49 days and a larval development period of 120 days (Romero et al., 2004); this long spawning time and prolonged planktonic larval phase may facilitate gene flow among distant populations by means of current‐driven dispersal, a phenomenon observed in other species having long larval periods. For example, Gomez‐Uchida et al. (2003) observed that, when coupled with the current coastal system off Chile, the 60‐day larval stage required by *Cancer setosus* promotes gene flow through effective larval interchange among geographically separated populations. Likewise, Cárdenas, Castilla, & Viard, 2016; Cárdenas, Silva, et al., 2009 found that the 90‐day period of pelagic larval duration in *Concholepas concholepas* (Disalvo, 1988) might explain the high connectivity observed for this species across the Peruvian–Chilean province, which even spans the recognized biogeographical boundaries at 30 S and 42 S. It is therefore possible to consider that passive and efficient larval dispersion by means of oceanic currents is homogenizing the genetic composition of *T. chocolata* populations around the Peruvian coast, even if the extent of adult movement is limited.

The unimodal mismatch distribution, significantly negative values obtained for Fu's Fs and Tajima's D, and BSP results all strongly support a historical expansion of *T. chocolata* along the Peruvian coastline. Although we could not accurately estimate the timing of demographic expansion because we lacked robust estimates of mutation rate, our results suggest that *T. chocolata* experienced two waves of population expansion. Analysis of the *CO1* dataset indicated the first expansion pulse to have taken place at around 800,000–700,000 years BP, followed by a more pronounced expansion at 400000 years BP. The latter expansion aligns with the MIS‐11 interglacial, a distinctly long and warm interglacial episode that occurred during the Pleistocene (Berger et al., 2016). However, our estimation of the onset of population expansion in *T. chocolata* differs from those reported for pelagic species inhabiting the South Pacific, such as the limpet *Siphonaria lessonii* (Pardo‐Gandarillas et al., 2018) and the cephalopod *Doryteuthis gahi* (Ibáñez et al., 2012). In those species, mtDNA patterns were consistent with large population expansions posterior to the LGM, the period of greatest ice advance. The discrepancy in our results could be because the western equatorial Pacific during the LGM was only about 3°C below modern temperatures (Rein et al., 2005; Tudhope et al., 2001), a climatic shift that might not have been strong enough to trigger a population expansion in *T. chocolata*. By contrast, the MIS‐11 featured extreme changes in temperature and sea level. Indeed, historical demography patterns similar to present findings in *T. chocolata* have been observed in rocky‐shore species from the northeastern Pacific, such as the mollusks *Mytilus californianus* and *Katharina tunicata*, and the crustacean *Balanus glandula* (Marko et al., 2010); in species from the northwestern Pacific such as the gastropod *Thais clavigera* (Guo et al., 2015); and in codistributed rocky‐shore species such as *Concholepas concholepas* (Cárdenas, Castilla, et al., 2016; Cárdenas, Silva, et al., 2009). Hence, events predating the LGM seem to have been more critical in shaping the demographic histories of rocky‐shore species (Marko et al., 2010).

The first wave of population expansion determined for *T. chocolata*, at around 800,000–700,000 years BP, also coincides with the most extreme low and high stands of the mid‐Pleistocene Revolution (Scourse, 2013). By 800,000 years BP, glacial cycles with sea level amplitudes of more than 100 m were occurring, mostly with periods on the order of 100,000 years (Berger et al., 2016). During these glacial–interglacial cycles, significant eustatic movements took place, including sea‐level lowering of at least several tens of meters during cold events and up to 100–140 m during full glacial episodes (Rabassa et al., 2005). These caused the South American coastlines to move westward by 100 km between 7° and9° S and by 20 km at 4°40′ S, resulting in the exposure of a large part of the South Pacific continental shelf (Ortlieb & Machare, 1989). Such substantial marine regressions probably caused habitat expansions for benthic invertebrates such as *T. chocolata*. In fact, DeVries (2007) showed that the smooth, refractory, chocolate‐brown outer shell layer of *T. chocolata* emerged as coastal water was cooling, submergent shorelines were rising, eustatic sea level was undergoing rapid and marked changes, and Peruvian shorelines were becoming straighter and more exposed. Moreover, Valdovinos et al. (2003) showed that the relative importance of factors controlling species diversity might have shifted in high latitudes along the southeastern Pacific, with available shelf area playing a more prominent role than temperature (Valdovinos et al., 2003). Hence, the first population expansion observed in *T. chocolata* could have occurred on account of the exposure of new rocky terrain belonging to the continental shelf; this could have favored *T. chocolata* and made it less vulnerable to changes in sea level.

It is worth motioning that the sudden population expansion observed in *T. chocolata* could have followed a genetic bottleneck or founder effect; however, with our current data, this scenario can be rejected as a hypothesis since for that sequence of events to have happened, the haplotypes present in the expanding low‐density peripheral (leading‐edge) populations would have to be shared with sites at the center of the distribution, even if said haplotypes are absent from marginal locations (Maggs et al., 2008). Leading‐edge populations are those whose demographic and allelic composition stochasticity are predictably stronger (Robalo et al., 2020) and thus are typically affected by founder events. Also, in the wake of a bottleneck or founder event, the geographic space becomes structured into regions of low diversity separated by sharp frequency gradients (Excoffier & Ray, 2008; Ibáñez et al., 2011; Robalo et al., 2020). In our datasets, haplotypes present in the leading‐edge patches were not shared with sites at the center of the distribution, and high haplotypic and nucleotide diversity values were observed for all sites. *T. chocolata* therefore seems to have retained exceptional levels of genetic diversity through its historical demographic expansions, an event that seems not consistent with a genetic bottleneck or founder effect.

Overall, our demographic time estimates should be tempered by consideration of the potential effects of three important sources of uncertainty: (a) evolutionary variability around coalescence; (b) inadequate sampling; and (c) uncertainty of the molecular clocks for *CO1* and 16S rRNA (Fernández‐Iriarte et al., 2020). Among these, we recognize evolutionary variability around the coalescence as the main source of uncertainty. Incomplete sampling of haplotypes was also inevitable. As our rarefaction curve showed, more individuals needed to be sampled in order to have a complete picture of haplotype distributions. Finally, when performing demographic inference analyses, the substitutional rate greatly determines the time estimation of population growth (Fernández‐Iriarte et al., 2020). Thus, the exact timing of demographic changes could be in error if the actual mutation rates of the taxa considered here were substantially larger than our 1.57% My^−1^‐based rate (Marko et al., 2010). Nonetheless, our findings regarding demographic population expansion are supported by the fossil records of *T. chocolata*. In Southern Peru, *T. chocolata* was found in the uppermost Pliocene, lower Pleistocene, and middle Pleistocene deposits of the Taime formation and marine terraces. DeVries (2007) also determined that *Thaisella* populations evolved rapidly in the southeastern Pacific Ocean at the beginning of the late Pliocene, concurrent with the start of an extinction event that within a million years eliminated 80% of molluscan species from the early Pliocene precursor to the modern Peruvian faunal province. *T. chocolata* fossils have also been dated to the latest part of the Pliocene, prior to when eustatic sea‐level changes and coastal uplift together began to produce marine terraces (DeVries, 2007). Our estimations, therefore, allow us to infer that demographic events in *T. chocolata* history are associated with the lower and middle Pleistocene, which agree with fossil records of the species.

Finally, the baseline genetic information gathered here may help improve the sustainable management of *T. chocolata*. In the first place, the presence of a genetically homogeneous population indicates that *T. chocolata* can be considered a single management unit, based on the concept that genetically distinct stocks, the basic unit for harvest and management, need to be managed as separate units (Cossu et al., 2021; Laikre et al., 2005). The genetic identification of the stock structure, however, is only the first step in implementing a sustainable management plan. Fishery managers should also consider estimating population abundance and population dynamics rates to reach appropriate levels of fishing mortality to maintain population sustainability and productivity (Cochrane, 2002). In fact, the Code of Conduct for Responsible Fisheries specifies that the states should adopt appropriate measures, based on the best scientific evidence available, to maintain or restore stocks at levels capable of producing maximum sustainable yield (FAO, 1995). If experimental data cannot be obtained to reach this objective, species dynamics can be characterized using individual‐based simulations such as those implemented by Strand (2002) and already applied to fisheries management (IWC, 2007). These simulations represent natural conditions in an explicitly stochastic fashion by treating individuals as autonomous units that move through the environment, experience demographic transitions, and interact with each other based upon computer‐generated random numbers (Strand, 2002). Managers should also consider evaluating the impacts of the changes in any of the ecosystem's biological, chemical, or physical components on the resource population and community (Cochrane, 2002). For example, in some South American countries, the use of butyltin (BT), an organotin compound used in antifouling paints, has not been regulated even though it was banned by the International Maritime Organization because it triggers imposex (IMO, 2000). Imposex is a type of sexual abnormality wherein male sex organs develop on female gastropods (Zou, 2019). Although BT causes female infertility and inhibits the release of capsules (often containing eggs) (Gibbs & Bryan, 1986; Stroben et al., 1995), no local regulatory strategies related to the use of BT‐based antifouling paints have been implemented in Peru, a country where the presence of BT in coastal areas has already been shown to alter the populations of *T. chocolata* inhabiting major fishing ports and national natural reserves (Castro et al., 2018; Castro & Fillmann, 2012; Chumbimune‐Ilizarbe & Ponce‐Mora, 2015). Ultimately, management plans should include threshold criteria for detecting biologically significant changes (Flanagan et al., 2018). A practical approach could consist of set trigger points throughout the range of an indicator variable to ensure that management action is initiated before a crisis point is reached. For example, Flanagan et al. (2018) suggest that a continuous decline in allelic richness at putatively adaptive *loci* or observation of low survival or fecundity over multiple sampling periods may trigger a management intervention such as genetic rescue to increase allelic richness or fitness. Achieving sustainable management, therefore, will require a proactive approach to seeking ways to optimize the benefits derived from the resource.

## 5 CONCLUSIONS

Measuring dispersal based on genetic patterns is essential to conservation and species management, as dispersal directly determines a species' response to selection and its capacity to respond to disturbances in a changing environment (Bradbury & Bentzen, 2007). Determining dispersal pathways through an understanding of genetic connectivity is, therefore, an essential prerequisite for devising effective fishery management strategies. Based on the analysis of two mitochondrial markers, *CO1* and 16S rRNA, the present study examined the genetic connectivity of the gastropod *T. chocolata* across the Peruvian coastal region. Our findings support the existence of a genetically homogeneous population within the survey area, which reconciles biological and management criteria. Nevertheless, as population genetic inferences based only on mtDNA can be misleading (Cárdenas et al., 2016; Cárdenas, Castilla, et al., 2016; Cárdenas, Silva, et al., 2009), these results must be treated with caution. For example, besides population expansion, negative values of Tajima's D can reflect other demographic scenarios such as selective sweeps. In both cases, the lineages in the genealogy are forced to coalesce at the time of the selective sweep or bottleneck—the average number of pairwise differences is decreased compared to the number of segregating sites, leading to negative Tajima's D values (Nielsen, 2001). It is therefore difficult to distinguish both scenarios when a single *locus* is considered. Using additional markers, a selective sweep in mtDNA can be differentiated from a demographic expansion if a low diversity in the whole genome is revealed (Ibáñez et al., 2011). Hence, further analyses using nuclear DNA markers or whole‐genome analysis could provide a complete perspective on population demography for this species. The accumulation of biological and ecological data also remains crucial, as such data are needed to determine the extent of migration, which will help to better define the mode of gene flow between populations (Sekino & Hara, 2001). Demographic history analyses in the present work also revealed that *T. chocolata* underwent two waves of population expansion, most likely during the lower and middle Pleistocene epoch. According to our data, *T. chocolata* retained exceptional genetic diversity levels following population expansion. This finding could provide helpful information for understanding this species' response to future climate change. The analysis of population connectivity in *T. chocolata*, therefore, can help establish a genetic baseline for this species in order to better maintain the equilibrium of the population while it is subjected to reasonable exploitation.

## AUTHOR CONTRIBUTIONS

**Eliana Zelada‐Mázmela:** Conceptualization (equal); project administration (equal); supervision (equal); writing – review and editing (supporting). **Lorenzo E. Reyes‐Flores:** Conceptualization (equal); data curation (equal); formal analysis (equal); investigation (equal); methodology (equal); project administration (equal); supervision (equal); validation (equal); writing – review and editing (supporting). **Julissa J. Sánchez‐Velásquez:** Data curation (equal); formal analysis (equal); methodology (equal); validation (equal); writing – original draft (lead); writing – review and editing (lead). **Claudia Ingar:** Investigation (equal); resources (equal). **Luis E. Santos‐Rojas:** Investigation (equal); resources (equal).

## FUNDING INFORMATION

This research did not receive any specific grant from the public, commercial, or not‐for‐profit funding agencies.

## CONFLICTS OF INTEREST

The authors report no conflicts of interest.

## Supporting information


Tables S1–S2

## Data Availability

The data that support the findings of this study are openly available in the GenBank database (www.ncbi.nlm.nih.gov/genbank) under accession numbers OK087138‐OK087293 and OK094932‐OK095087. CO1 and 16S rRNA genomic datasets used during this study are also stored and available at https://doi.org/10.5061/dryad.jsxksn0d1.
