# Supplementary material for: Population structure and demographic history of the gastropod Thaisella chocolata (Duclos, 1832) from the Southeast Pacific inferred from mitochondrial DNA analyses
Source: Ecol Evol. 2022 Sep 9;12(9):e9276. doi: 10.1002/ece3.9276 (PMC9463045; doi:10.1002/ece3.9276)
Supplement: Supplementary file 1 — Tables S1–S2 [file ECE3-12-e9276-s001.docx]

**Supplementary Materials**

**Table S1.** Sample information including locality, collection codes, date of collection, and GenBank accession number for the *CO1* genomic dataset used in the present study.

| **Species; locality; collection code, date** | **GenBank ID** |
| --- | --- |
| *Thaisella chocolata*; Bayovar, Piura, Peru; LGFYR.By2; 8 Sep. 2019 | OK087138 |
| *T. chocolata*; Bayovar, Piura, Peru; LGFYR.By3; 8 Sep. 2019 | OK087139 |
| *T. chocolata*; Bayovar, Piura, Peru; LGFYR.By4; 8 Sep. 2019 | OK087140 |
| *T. chocolata*; Bayovar, Piura, Peru; LGFYR.By5; 8 Sep. 2019 | OK087141 |
| *T. chocolata*; Bayovar, Piura, Peru; LGFYR.By6; 8 Sep. 2019 | OK087142 |
| *T. chocolata*; Bayovar, Piura, Peru; LGFYR.By7; 8 Sep. 2019 | OK087143 |
| *T. chocolata*; Bayovar, Piura, Peru; LGFYR.By8; 8 Sep. 2019 | OK087144 |
| *T. chocolata*; Bayovar, Piura, Peru; LGFYR.By9; 8 Sep. 2019 | OK087145 |
| *T. chocolata*; Bayovar, Piura, Peru; LGFYR.By10; 8 Sep. 2019 | OK087146 |
| *T. chocolata*; Bayovar, Piura, Peru; LGFYR.By11; 8 Sep. 2019 | OK087147 |
| *T. chocolata*; Bayovar, Piura, Peru; LGFYR.By12; 8 Sep. 2019 | OK087148 |
| *T. chocolata*; Bayovar, Piura, Peru; LGFYR.By14; 8 Sep. 2019 | OK087149 |
| *T. chocolata*; Bayovar, Piura, Peru; LGFYR.By15; 8 Sep. 2019 | OK087150 |
| *T. chocolata*; Bayovar, Piura, Peru; LGFYR.By16; 8 Sep. 2019 | OK087151 |
| *T. chocolata*; Bayovar, Piura, Peru; LGFYR.By17; 8 Sep. 2019 | OK087152 |
| *T. chocolata*; Bayovar, Piura, Peru; LGFYR.By18; 8 Sep. 2019 | OK087153 |
| *T. chocolata*; Bayovar, Piura, Peru; LGFYR.By19; 8 Sep. 2019 | OK087154 |
| *T. chocolata*; Bayovar, Piura, Peru; LGFYR.By29; 8 Sep. 2019 | OK087155 |
| *T. chocolata*; Bayovar, Piura, Peru; LGFYR.By21; 8 Sep. 2019 | OK087156 |
| *T. chocolata*; Bayovar, Piura, Peru; LGFYR.By22; 8 Sep. 2019 | OK087157 |
| *T. chocolata*; Pimentel, Lambayeque, Peru; LGFYR.Pm1; 11 Jan. 2020 | OK087158 |
| *T. chocolata*; Pimentel, Lambayeque, Peru; LGFYR.Pm2; 11 Jan. 2020 | OK087159 |
| *T. chocolata*; Pimentel, Lambayeque, Peru; LGFYR.Pm3; 11 Jan. 2020 | OK087160 |
| *T. chocolata*; Pimentel, Lambayeque, Peru; LGFYR.Pm4; 11 Jan. 2020 | OK087161 |
| *T. chocolata*; Pimentel, Lambayeque, Peru; LGFYR.Pm5; 11 Jan. 2020 | OK087162 |
| *T. chocolata*; Pimentel, Lambayeque, Peru; LGFYR.Pm6; 11 Jan. 2020 | OK087163 |
| *T. chocolata*; Pimentel, Lambayeque, Peru; LGFYR.Pm7; 11 Jan. 2020 | OK087164 |
| *T. chocolata*; Pimentel, Lambayeque, Peru; LGFYR.Pm8; 11 Jan. 2020 | OK087165 |
| *T. chocolata*; Pimentel, Lambayeque, Peru; LGFYR.Pm9; 11 Jan. 2020 | OK087166 |
| *T. chocolata*; Pimentel, Lambayeque, Peru; LGFYR.Pm10; 11 Jan. 2020 | OK087167 |
| *T. chocolata*; Pimentel, Lambayeque, Peru; LGFYR.Pm11; 11 Jan. 2020 | OK087168 |
| *T. chocolata*; Pimentel, Lambayeque, Peru; LGFYR.Pm12; 11 Jan. 2020 | OK087169 |
| *T. chocolata*; Pimentel, Lambayeque, Peru; LGFYR.Pm13; 11 Jan. 2020 | OK087170 |
| *T. chocolata*; Pimentel, Lambayeque, Peru; LGFYR.Pm14; 11 Jan. 2020 | OK087171 |
| *T. chocolata*; Pimentel, Lambayeque, Peru; LGFYR.Pm15; 11 Jan. 2020 | OK087172 |
| *T. chocolata*; Pimentel, Lambayeque, Peru; LGFYR.Pm16; 11 Jan. 2020 | OK087173 |
| *T. chocolata*; Pimentel, Lambayeque, Peru; LGFYR.Pm17; 11 Jan. 2020 | OK087174 |
| *T. chocolata*; Pimentel, Lambayeque, Peru; LGFYR.Pm18; 11 Jan. 2020 | OK087175 |
| *T. chocolata*; Pimentel, Lambayeque, Peru; LGFYR.Pm20; 11 Jan. 2020 | OK087176 |
| *T. chocolata*; Pimentel, Lambayeque, Peru; LGFYR.Pm22; 11 Jan. 2020 | OK087177 |
| *T. chocolata*; Salaverry, La Libertad, Peru; LGFYR.Sl_3; 11 Jan. 2020 | OK087178 |
| *T. chocolata*; Salaverry, La Libertad, Peru; LGFYR.Sl_4; 11 Jan. 2020 | OK087179 |
| *T. chocolata*; Salaverry, L Libertad, Peru; LGFYR.Sl_5; 11 Jan. 2020 | OK087180 |

**Table S1.** **Continue**

| **Species; locality; collection code, date** | **GenBank ID** |
| --- | --- |
| *T. chocolata*; Salaverry, La Libertad, Peru, LGFYR.Sl_6; 11 Jan. 2020 | OK087181 |
| *T. chocolata*; Salaverry, La Libertad, Peru; LGFYR.Sl_7; 11 Jan. 2020 | OK087182 |
| *T. chocolata*; Salaverry, La Libertad, Peru; LGFYR.Sl_8; 11 Jan. 2020 | OK087183 |
| *T. chocolata*; Salaverry, La Libertad, Peru; LGFYR.Sl_9; 11 Jan. 2020 | OK087184 |
| *T. chocolata*; Salaverry, La Libertad, Peru; LGFYR.Sl_10; 11 Jan. 2020 | OK087185 |
| *T. chocolata*; Salaverry, La Libertad, Peru; LGFYR.Sl_11; 11 Jan. 2020 | OK087186 |
| *T. chocolata*; Salaverry, La Libertad, Peru; LGFYR.Sl_12; 11 Jan. 2020 | OK087187 |
| *T. chocolata*; Salaverry, La Libertad, Peru; LGFYR.Sl_13; 11 Jan. 2020 | OK087188 |
| *T. chocolata*; Salaverry, La Libertad, Peru; LGFYR.Sl_14; 11 Jan. 2020 | OK087189 |
| *T. chocolata*; Salaverry, La Libertad, Peru; LGFYR.Sl_15; 11 Jan. 2020 | OK087190 |
| *T. chocolata*; Salaverry, La Libertad, Peru; LGFYR.Sl_16; 11 Jan. 2020 | OK087191 |
| *T. chocolata*; Salaverry, La Libertad, Peru; LGFYR.Sl_17; 11 Jan. 2020 | OK087192 |
| *T. chocolata*; Salaverry, La Libertad, Peru; LGFYR.Sl_19; 11 Jan. 2020 | OK087193 |
| *T. chocolata*; Salaverry, La Libertad, Peru; LGFYR.Sl_22; 11 Jan. 2020 | OK087194 |
| *T. chocolata*; Salaverry, La Libertad, Peru; LGFYR.Sl_23; 11 Jan. 2020 | OK087195 |
| *T. chocolata*; Chao, La Libertad, Peru; LGFYR.Ch2; 11 Jan. 2020 | OK087196 |
| *T. chocolata*; Chao, La Libertad, Peru; LGFYR.Ch3; 11 Jan. 2020 | OK087197 |
| *T. chocolata*; Chao, La Libertad, Peru; LGFYR.Ch4; 11 Jan. 2020 | OK087198 |
| *T. chocolata*; Chao, La Libertad, Peru; LGFYR.Ch5; 11 Jan. 2020 | OK087199 |
| *T. chocolata*; Chao, La Libertad, Peru; LGFYR.Ch6; 11 Jan. 2020 | OK087200 |
| *T. chocolata*; Chao, La Libertad, Peru; LGFYR.Ch7; 11 Jan. 2020 | OK087201 |
| *T. chocolata*; Chao, La Libertad, Peru; LGFYR.Ch8; 11 Jan. 2020 | OK087202 |
| *T. chocolata*; Chao, La Libertad, Peru; LGFYR.Ch9; 11 Jan. 2020 | OK087203 |
| *T. chocolata*; Chao, La Libertad, Peru; LGFYR.Ch10; 11 Jan. 2020 | OK087204 |
| *T. chocolata*; Chao, La Libertad, Peru; LGFYR.Ch11; 11 Jan. 2020 | OK087205 |
| *T. chocolata*; Chao, La Libertad, Peru; LGFYR.Ch12; 11 Jan. 2020 | OK087206 |
| *T. chocolata*; Chao, La Libertad, Peru; LGFYR.Ch13; 11 Jan. 2020 | OK087207 |
| *T. chocolata*; Chao, La Libertad, Peru; LGFYR.Ch14; 11 Jan. 2020 | OK087208 |
| *T. chocolata*; Chao, La Libertad, Peru; LGFYR.Ch15; 11 Jan. 2020 | OK087209 |
| *T. chocolata*; Chao, La Libertad, Peru; LGFYR.Ch16; 11 Jan. 2020 | OK087210 |
| *T. chocolata*; Chao, La Libertad, Peru; LGFYR.Ch17; 11 Jan. 2020 | OK087211 |
| *T. chocolata*; Chao, La Libertad, Peru; LGFYR.Ch18; 11 Jan. 2020 | OK087212 |
| *T. chocolata*; Chao, La Libertad, Peru; LGFYR.Ch19; 11 Jan. 2020 | OK087213 |
| *T. chocolata*; Chao, La Libertad, Peru; LGFYR.Ch20; 11 Jan. 2020 | OK087214 |
| *T. chocolata*; Chao, La Libertad, Peru; LGFYR.Ch22; 11 Jan. 2020 | OK087215 |
| *T. chocolata*; Samanco, Ancash, Peru; LGFYR.Sm1; 30 Nov. 2019 | OK087216 |
| *T. chocolata*; Samanco, Ancash, Peru; LGFYR.Sm2; 30 Nov. 2019 | OK087217 |
| *T. chocolata*; Samanco, Ancash, Peru; LGFYR.Sm3; 30 Nov. 2019 | OK087218 |
| *T. chocolata*; Samanco, Ancash, Peru; LGFYR.Sm4; 30 Nov. 2019 | OK087219 |
| *T. chocolata*; Samanco, Ancash, Peru; LGFYR.Sm5; 30 Nov. 2019 | OK087220 |
| *T. chocolata*; Samanco, Ancash, Peru; LGFYR.Sm6; 30 Nov. 2019 | OK087221 |
| *T. chocolata*; Samanco, Ancash, Peru; LGFYR.Sm7; 30 Nov. 2019 | OK087222 |
| *T. chocolata*; Samanco, Ancash, Peru; LGFYR.Sm8; 30 Nov. 2019 | OK087223 |
| *T. chocolata*; Samanco, Ancash, Peru; LGFYR.Sm9; 30 Nov. 2019 | OK087224 |
| *T. chocolata*; Samanco, Ancash, Peru; LGFYR.Sm10; 30 Nov. 2019 | OK087225 |
| *T. chocolata*; Samanco, Ancash, Peru; LGFYR.Sm11; 30 Nov. 2019 | OK087226 |

**Table S1.** **Continue**

| **Species; locality; collection code, date** | **GenBank ID** |
| --- | --- |
| *T. chocolata*; Samanco, Ancash, Peru; LGFYR.Sm12; 30 Nov. 2019 | OK087227 |
| *T. chocolata*; Samanco, Ancash, Peru; LGFYR.Sm13; 30 Nov. 2019 | OK087228 |
| *T. chocolata*; Samanco, Ancash, Peru; LGFYR.Sm14; 30 Nov. 2019 | OK087229 |
| *T. chocolata*; Samanco, Ancash, Peru; LGFYR.Sm15; 30 Nov. 2019 | OK087230 |
| *T. chocolata*; Samanco, Ancash, Peru; LGFYR.Sm16; 30 Nov. 2019 | OK087231 |
| *T. chocolata*; Samanco, Ancash, Peru; LGFYR.Sm20; 30 Nov. 2019 | OK087232 |
| *T. chocolata*; Samanco, Ancash, Peru; LGFYR.Sm23; 30 Nov. 2019 | OK087233 |
| *T. chocolata*; Samanco, Ancash, Peru; LGFYR.Sm24; 30 Nov. 2019 | OK087234 |
| *T. chocolata*; Chorrillos, Lima, Peru; LGFYR.Lm3; 30 Nov. 2019 | OK087235 |
| *T. chocolata*; Chorrillos, Lima, Peru; LGFYR.Lm4; 24 Feb. 2020 | OK087236 |
| *T. chocolata*; Chorrillos, Lima, Peru; LGFYR.Lm6; 24 Feb. 2020 | OK087237 |
| *T. chocolata*; Chorrillos, Lima, Peru; LGFYR.Lm8; 24 Feb. 2020 | OK087238 |
| *T. chocolata*; Chorrillos, Lima, Peru; LGFYR.Lm9; 24 Feb. 2020 | OK087239 |
| *T. chocolata*; Chorrillos, Lima, Peru; LGFYR.Lm10; 24 Feb. 2020 | OK087240 |
| *T. chocolata*; Chorrillos, Lima, Peru; LGFYR.Lm12; 24 Feb. 2020 | OK087241 |
| *T. chocolata*; Chorrillos, Lima, Peru; LGFYR.Lm13; 24 Feb. 2020 | OK087242 |
| *T. chocolata*; Chorrillos, Lima, Peru; LGFYR.Lm14; 24 Feb. 2020 | OK087243 |
| *T. chocolata*; Chorrillos, Lima, Peru; LGFYR.Lm15; 24 Feb. 2020 | OK087244 |
| *T. chocolata*; Chorrillos, Lima, Peru; LGFYR.Lm16; 24 Feb. 2020 | OK087245 |
| *T. chocolata*; Chorrillos, Lima, Peru; LGFYR.Lm17; 24 Feb. 2020 | OK087246 |
| *T. chocolata*; Chorrillos, Lima, Peru; LGFYR.Lm18; 24 Feb. 2020 | OK087247 |
| *T. chocolata*; Chorrillos, Lima, Peru; LGFYR.Lm19; 24 Feb. 2020 | OK087248 |
| *T. chocolata*; Chorrillos, Lima, Peru; LGFYR.Lm20; 24 Feb. 2020 | OK087249 |
| *T. chocolata*; Chorrillos, Lima, Peru; LGFYR.Lm21; 24 Feb. 2020 | OK087250 |
| *T. chocolata*; Chorrillos, Lima, Peru; LGFYR.Lm22; 24 Feb. 2020 | OK087251 |
| *T. chocolata*; Chorrillos, Lima, Peru; LGFYR.Lm23; 24 Feb. 2020 | OK087252 |
| *T. chocolata*; Chorrillos, Lima, Peru; LGFYR.Lm24; 24 Feb. 2020 | OK087253 |
| *T. chocolata*; Pisco, Ica, Peru; LGFYR.Ps1; 22 Sep. 2018 | OK087254 |
| *T. chocolata*; Pisco, Ica, Peru; LGFYR.Ps2; 22 Sep. 2018 | OK087255 |
| *T. chocolata*; Pisco, Ica, Peru; LGFYR.Ps3; 22 Sep. 2018 | OK087256 |
| *T. chocolata*; Pisco, Ica, Peru; LGFYR.Ps4; 22 Sep. 2018 | OK087257 |
| *T. chocolata*; Pisco, Ica, Peru; LGFYR.Ps5; 22 Sep. 2018 | OK087258 |
| *T. chocolata*; Pisco, Ica, Peru; LGFYR.Ps6; 22 Sep. 2018 | OK087259 |
| *T. chocolata*; Pisco, Ica, Peru; LGFYR.Ps7; 22 Sep. 2018 | OK087260 |
| *T. chocolata*; Pisco, Ica, Peru; LGFYR.Ps8; 22 Sep. 2018 | OK087261 |
| *T. chocolata*; Pisco, Ica, Peru; LGFYR.Ps9; 22 Sep. 2018 | OK087262 |
| *T. chocolata*; Pisco, Ica, Peru; LGFYR.Ps10; 22 Sep. 2018 | OK087263 |
| *T. chocolata*; Pisco, Ica, Peru; LGFYR.Ps11; 22 Sep. 2018 | OK087264 |
| *T. chocolata*; Pisco, Ica, Peru; LGFYR.Ps12; 22 Sep. 2018 | OK087265 |
| *T. chocolata*; Pisco, Ica, Peru; LGFYR.Ps13; 22 Sep. 2018 | OK087266 |
| *T. chocolata*; Pisco, Ica, Peru; LGFYR.Ps14; 22 Sep. 2018 | OK087267 |
| *T. chocolata*; Pisco, Ica, Peru; LGFYR.Ps15; 22 Sep. 2018 | OK087268 |
| *T. chocolata*; Pisco, Ica, Peru; LGFYR.Ps16; 22 Sep. 2018 | OK087269 |
| *T. chocolata*; Pisco, Ica, Peru; LGFYR.Ps17; 22 Sep. 2018 | OK087270 |
| *T. chocolata*; Pisco, Ica, Peru; LGFYR.Ps18; 22 Sep. 2018 | OK087271 |
| *T. chocolata*; Pisco, Ica, Peru; LGFYR.Ps19; 22 Sep. 2018 | OK087272 |

**Table S1.** **Continue**

| **Species; locality; collection code, date** | **GenBank ID** |
| --- | --- |
| *T. chocolata*; Pisco, Ica, Peru; LGFYR.Ps20; 22 Sep. 2018 | OK087273 |
| *T. chocolata*; Pisco, Ica, Peru; LGFYR.Ps21; 22 Sep. 2018 | OK087274 |
| *T. chocolata*; Pisco, Ica, Peru; LGFYR.Ps21; 22 Sep. 2018 | OK087275 |
| *T. chocolata*; Marcona, Ica, Peru; LGFYR.Mc1; 19 Mar. 2019 | OK087276 |
| *T. chocolata*; Marcona, Ica, Peru; LGFYR.Mc2; 19 Mar. 2019 | OK087277 |
| *T. chocolata*; Marcona, Ica, Peru; LGFYR.Mc3; 19 Mar. 2019 | OK087278 |
| *T. chocolata*; Marcona, Ica, Peru; LGFYR.Mc4; 19 Mar. 2019 | OK087279 |
| *T. chocolata*; Marcona, Ica, Peru; LGFYR.Mc5; 19 Mar. 2019 | OK087280 |
| *T. chocolata*; Marcona, Ica, Peru; LGFYR.Mc6; 19 Mar. 2019 | OK087281 |
| *T. chocolata*; Marcona, Ica, Peru; LGFYR.Mc7; 19 Mar. 2019 | OK087282 |
| *T. chocolata*; Marcona, Ica, Peru; LGFYR.Mc8; 19 Mar. 2019 | OK087283 |
| *T. chocolata*; Marcona, Ica, Peru; LGFYR.Mc9; 19 Mar. 2019 | OK087284 |
| *T. chocolata*; Marcona, Ica, Peru; LGFYR.Mc10; 19 Mar. 2019 | OK087285 |
| *T. chocolata*; Marcona, Ica, Peru; LGFYR.Mc11; 19 Mar. 2019 | OK087286 |
| *T. chocolata*; Marcona, Ica, Peru; LGFYR.Mc12; 19 Mar. 2019 | OK087287 |
| *T. chocolata*; Marcona, Ica, Peru; LGFYR.Mc13; 19 Mar. 2019 | OK087288 |
| *T. chocolata*; Marcona, Ica, Peru; LGFYR.Mc14; 19 Mar. 2019 | OK087289 |
| *T. chocolata*; Marcona, Ica, Peru; LGFYR.Mc15; 19 Mar. 2019 | OK087290 |
| *T. chocolata*; Marcona, Ica, Peru; LGFYR.Mc17; 19 Mar. 2019 | OK087291 |
| *T. chocolata*; Marcona, Ica, Peru; LGFYR.Mc21; 19 Mar. 2019 | OK087292 |
| *T. chocolata*; Marcona, Ica, Peru; LGFYR.Mc22; 19 Mar. 2019 | OK087293 |
| *Stramonita biserialis*; Cañete, Lima, Peru; LGFYR.Sbi.317.5; 17 Apr. 2017 | OM338107 |
| *S. biserialis*; Santa, Ancash, Peru; LGFYR.Sbi.329.3; 30 Aug. 2017 | OM338108 |
| *S. biserialis*; Santa, Ancash, Peru; LGFYR.Sbi.329.7; 30 Aug. 2017 | OM338109 |

**Table S2.** Sample information including locality, collectors name, collection codes, date of collection, and GenBank accession number for the 16S rRNA genomic dataset used in the present study.

| **Species; locality; collectors name; collection code, date** | **GenBank ID** |
| --- | --- |
| *Thaisella chocolata*; Bayovar, Piura, Peru; LGFYR.By2; 8 Sep. 2019 | OK094932 |
| *T. chocolata*; Bayovar, Piura, Peru; LGFYR.By3; 8 Sep. 2019 | OK094933 |
| *T. chocolata*; Bayovar, Piura, Peru; LGFYR.By4; 8 Sep. 2019 | OK094934 |
| *T. chocolata*; Bayovar, Piura, Peru; LGFYR.By5; 8 Sep. 2019 | OK094935 |
| *T. chocolata*; Bayovar, Piura, Peru; LGFYR.By6; 8 Sep. 2019 | OK094936 |
| *T. chocolata*; Bayovar, Piura, Peru; LGFYR.By7; 8 Sep. 2019 | OK094937 |
| *T. chocolata*; Bayovar, Piura, Peru; LGFYR.By8; 8 Sep. 2019 | OK094938 |
| *T. chocolata*; Bayovar, Piura, Peru; LGFYR.By9; 8 Sep. 2019 | OK094939 |
| *T. chocolata*; Bayovar, Piura, Peru; LGFYR.By10; 8 Sep. 2019 | OK094940 |
| *T. chocolata*; Bayovar, Piura, Peru; LGFYR.By11; 8 Sep. 2019 | OK094941 |
| *T. chocolata*; Bayovar, Piura, Peru; LGFYR.By12; 8 Sep. 2019 | OK094942 |
| *T. chocolata*; Bayovar, Piura, Peru; LGFYR.By14; 8 Sep. 2019 | OK094943 |
| *T. chocolata*; Bayovar, Piura, Peru; LGFYR.By15; 8 Sep. 2019 | OK094944 |
| *T. chocolata*; Bayovar, Piura, Peru; LGFYR.By16; 8 Sep. 2019 | OK094945 |
| *T. chocolata*; Bayovar, Piura, Peru; LGFYR.By17; 8 Sep. 2019 | OK094946 |
| *T. chocolata*; Bayovar, Piura, Peru; LGFYR.By18; 8 Sep. 2019 | OK094947 |
| *T. chocolata*; Bayovar, Piura, Peru; LGFYR.By19; 8 Sep. 2019 | OK094948 |
| *T. chocolata*; Bayovar, Piura, Peru; LGFYR.By20; 8 Sep. 2019 | OK094949 |
| *T. chocolata*; Bayovar, Piura, Peru; LGFYR.By21; 8 Sep. 2019 | OK094950 |
| *T. chocolata*; Bayovar, Piura, Peru; LGFYR.By22; 8 Sep. 2019 | OK094951 |
| *T. chocolata*; Pimentel, Lambayeque, Peru; LGFYR.Pm1; 11 Jan. 2020 | OK094952 |
| *T. chocolata*; Pimentel, Lambayeque, Peru; LGFYR.Pm2; 11 Jan. 2020 | OK094953 |
| *T. chocolata*; Pimentel, Lambayeque, Peru; LGFYR.Pm3; 11 Jan. 2020 | OK094954 |
| *T. chocolata*; Pimentel, Lambayeque, Peru; LGFYR.Pm4; 11 Jan. 2020 | OK094955 |
| *T. chocolata*; Pimentel, Lambayeque, Peru; LGFYR.Pm5; 11 Jan. 2020 | OK094956 |
| *T. chocolata*; Pimentel, Lambayeque, Peru; LGFYR.Pm6; 11 Jan. 2020 | OK094957 |
| *T. chocolata*; Pimentel, Lambayeque, Peru; LGFYR.Pm7; 11 Jan. 2020 | OK094958 |
| *T. chocolata*; Pimentel, Lambayeque, Peru; LGFYR.Pm8; 11 Jan. 2020 | OK094959 |
| *T. chocolata*; Pimentel, Lambayeque, Peru; LGFYR.Pm9; 11 Jan. 2020 | OK094960 |
| *T. chocolata*; Pimentel, Lambayeque, Peru; LGFYR.Pm10; 11 Jan. 2020 | OK094961 |
| *T. chocolata*; Pimentel, Lambayeque, Peru; LGFYR.Pm11; 11 Jan. 2020 | OK094962 |
| *T. chocolata*; Pimentel, Lambayeque, Peru; LGFYR.Pm12; 11 Jan. 2020 | OK094963 |
| *T. chocolata*; Pimentel, Lambayeque, Peru; LGFYR.Pm13; 11 Jan. 2020 | OK094964 |
| *T. chocolata*; Pimentel, Lambayeque, Peru; LGFYR.Pm14; 11 Jan. 2020 | OK094965 |
| *T. chocolata*; Pimentel, Lambayeque, Peru; LGFYR.Pm15; 11 Jan. 2020 | OK094966 |
| *T. chocolata*; Pimentel, Lambayeque, Peru; LGFYR.Pm16; 11 Jan. 2020 | OK094967 |
| *T. chocolata*; Pimentel, Lambayeque, Peru; LGFYR.Pm17; 11 Jan. 2020 | OK094968 |
| *T. chocolata*; Pimentel, Lambayeque, Peru; LGFYR.Pm18; 11 Jan. 2020 | OK094969 |
| *T. chocolata*; Pimentel, Lambayeque, Peru; LGFYR.Pm19; 11 Jan. 2020 | OK094970 |
| *T. chocolata*; Pimentel, Lambayeque, Peru; LGFYR.Pm22; 11 Jan. 2020 | OK094971 |
| *T. chocolata*; Salaverry, La Libertad, Peru; LGFYR.Sl_3; 11 Jan. 2020 | OK094972 |
| *T. chocolata*; Salaverry, La Libertad, Peru; LGFYR.Sl_4; 11 Jan. 2020 | OK094973 |
| *T. chocolata*; Salaverry, La Libertad, Peru; LGFYR.Sl_5; 11 Jan. 2020 | OK094974 |
| *T. chocolata*; Salaverry, La Libertad, Peru; LGFYR.Sl_6; 11 Jan. 2020 | OK094975 |
| *T. chocolata*; Salaverry, La Libertad, Peru; LGFYR.Sl_7; 11 Jan. 2020 | OK094976 |

**Table S2.** **Continue**

| **Species; locality; collectors name; collection code, date** | **GenBank ID** |
| --- | --- |
| *T. chocolata*; Salaverry, La Libertad, Peru; LGFYR.Sl_8; 11 Jan. 2020 | OK094977 |
| *T. chocolata*; Salaverry, La Libertad, Peru; LGFYR.Sl_9; 11 Jan. 2020 | OK094978 |
| *T. chocolata*; Salaverry, La Libertad, Peru; LGFYR.Sl_10; 11 Jan. 2020 | OK094979 |
| *T. chocolata*; Salaverry, La Libertad, Peru; LGFYR.Sl_11; 11 Jan. 2020 | OK094980 |
| *T. chocolata*; Salaverry, La Libertad, Peru; LGFYR.Sl_12; 11 Jan. 2020 | OK094981 |
| *T. chocolata*; Salaverry, La Libertad, Peru; LGFYR.Sl_13; 11 Jan. 2020 | OK094982 |
| *T. chocolata*; Salaverry, La Libertad, Peru; LGFYR.Sl_14; 11 Jan. 2020 | OK094983 |
| *T. chocolata*; Salaverry, La Libertad, Peru; LGFYR.Sl_15; 11 Jan. 2020 | OK094984 |
| *T. chocolata*; Salaverry, La Libertad, Peru; LGFYR.Sl_16; 11 Jan. 2020 | OK094985 |
| *T. chocolata*; Salaverry, La Libertad, Peru; LGFYR.Sl_17; 11 Jan. 2020 | OK094986 |
| *T. chocolata*; Salaverry, La Libertad, Peru; LGFYR.Sl_18; 11 Jan. 2020 | OK094987 |
| *T. chocolata*; Salaverry, La Libertad, Peru; LGFYR.Sl_22; 11 Jan. 2020 | OK094988 |
| *T. chocolata*; Salaverry, La Libertad, Peru; LGFYR.Sl_23; 11 Jan. 2020 | OK094989 |
| *T. chocolata*; Chao, La Libertad, Peru; LGFYR.Ch2; 11 Jan. 2020 | OK094990 |
| *T. chocolata*; Chao, La Libertad, Peru; LGFYR.Ch3; 11 Jan. 2020 | OK094991 |
| *T. chocolata*; Chao, La Libertad, Peru; LGFYR.Ch4; 11 Jan. 2020 | OK094992 |
| *T. chocolata*; Chao, La Libertad, Peru; LGFYR.Ch5; 11 Jan. 2020 | OK094993 |
| *T. chocolata*; Chao, La Libertad, Peru; LGFYR.Ch6; 11 Jan. 2020 | OK094994 |
| *T. chocolata*; Chao, La Libertad, Peru; LGFYR.Ch7; 11 Jan. 2020 | OK094995 |
| *T. chocolata*; Chao, La Libertad, Peru; LGFYR.Ch8; 11 Jan. 2020 | OK094996 |
| *T. chocolata*; Chao, La Libertad, Peru; LGFYR.Ch9; 11 Jan. 2020 | OK094997 |
| *T. chocolata*; Chao, La Libertad, Peru; LGFYR.Ch10; 11 Jan. 2020 | OK094998 |
| *T. chocolata*; Chao, La Libertad, Peru; LGFYR.Ch11; 11 Jan. 2020 | OK094999 |
| *T. chocolata*; Chao, La Libertad, Peru; LGFYR.Ch12; 11 Jan. 2020 | OK095000 |
| *T. chocolata*; Chao, La Libertad, Peru; LGFYR.Ch13; 11 Jan. 2020 | OK095001 |
| *T. chocolata*; Chao, La Libertad, Peru; LGFYR.Ch14; 11 Jan. 2020 | OK095002 |
| *T. chocolata*; Chao, La Libertad, Peru; LGFYR.Ch15; 11 Jan. 2020 | OK095003 |
| *T. chocolata*; Chao, La Libertad, Peru; LGFYR.Ch15; 11 Jan. 2020 | OK095004 |
| *T. chocolata*; Chao, La Libertad, Peru; LGFYR.Ch16; 11 Jan. 2020 | OK095005 |
| *T. chocolata*; Chao, La Libertad, Peru; LGFYR.Ch17; 11 Jan. 2020 | OK095006 |
| *T. chocolata*; Chao, La Libertad, Peru; LGFYR.Ch18; 11 Jan. 2020 | OK095007 |
| *T. chocolata*; Chao, La Libertad, Peru; LGFYR.Ch19; 11 Jan. 2020 | OK095008 |
| *T. chocolata*; Chao, La Libertad, Peru; LGFYR.Ch22; 11 Jan. 2020 | OK095009 |
| *T. chocolata*; Samanco, Ancash, Peru; LGFYR.Sm1; 30 Nov. 2019 | OK095010 |
| *T. chocolata*; Samanco, Ancash, Peru; LGFYR.Sm2; 30 Nov. 2019 | OK095011 |
| *T. chocolata*; Samanco, Ancash, Peru; LGFYR.Sm3; 30 Nov. 2019 | OK095012 |
| *T. chocolata*; Samanco, Ancash, Peru; LGFYR.Sm4; 30 Nov. 2019 | OK095013 |
| *T. chocolata*; Samanco, Ancash, Peru; LGFYR.Sm5; 30 Nov. 2019 | OK095014 |
| *T. chocolata*; Samanco, Ancash, Peru; LGFYR.Sm6; 30 Nov. 2019 | OK095015 |
| *T. chocolata*; Samanco, Ancash, Peru; LGFYR.Sm7; 30 Nov. 2019 | OK095016 |
| *T. chocolata*; Samanco, Ancash, Peru; LGFYR.Sm8; 30 Nov. 2019 | OK095017 |
| *T. chocolata*; Samanco, Ancash, Peru; LGFYR.Sm9; 30 Nov. 2019 | OK095018 |
| *T. chocolata*; Samanco, Ancash, Peru; LGFYR.Sm10; 30 Nov. 2019 | OK095019 |
| *T. chocolata*; Samanco, Ancash, Peru; LGFYR.Sm11; 30 Nov. 2019 | OK095020 |
| *T. chocolata*; Samanco, Ancash, Peru; LGFYR.Sm12; 30 Nov. 2019 | OK095021 |
| *T. chocolata*; Samanco, Ancash, Peru; LGFYR.Sm13; 30 Nov. 2019 | OK095022 |

**Table S2.** **Continue**

| **Species; locality; collectors name; collection code, date** | **GenBank ID** |
| --- | --- |
| *T. chocolata*; Samanco, Ancash, Peru; LGFYR.Sm14; 30 Nov. 2019 | OK095023 |
| *T. chocolata*; Samanco, Ancash, Peru; LGFYR.Sm15; 30 Nov. 2019 | OK095024 |
| *T. chocolata*; Samanco, Ancash, Peru; LGFYR.Sm16; 30 Nov. 2019 | OK095025 |
| *T. chocolata*; Samanco, Ancash, Peru; LGFYR.Sm21; 30 Nov. 2019 | OK095026 |
| *T. chocolata*; Samanco, Ancash, Peru; LGFYR.Sm23; 30 Nov. 2019 | OK095027 |
| *T. chocolata*; Samanco, Ancash, Peru; LGFYR.Sm24; 30 Nov. 2019 | OK095028 |
| *T. chocolata*; Chorrillos, Lima, Peru; LGFYR.Lm3; 24 Feb. 2020 | OK095029 |
| *T. chocolata*; Chorrillos, Lima, Peru; LGFYR.Lm4; 24 Feb. 2020 | OK095030 |
| *T. chocolata*; Chorrillos, Lima, Peru; LGFYR.Lm6; 24 Feb. 2020 | OK095031 |
| *T. chocolata*; Chorrillos, Lima, Peru; LGFYR.Lm8; 24 Feb. 2020 | OK095032 |
| *T. chocolata*; Chorrillos, Lima, Peru; LGFYR.Lm9; 24 Feb. 2020 | OK095033 |
| *T. chocolata*; Chorrillos, Lima, Peru; LGFYR.Lm10; 24 Feb. 2020 | OK095034 |
| *T. chocolata*; Chorrillos, Lima, Peru; LGFYR.Lm12; 24 Feb. 2020 | OK095035 |
| *T. chocolata*; Chorrillos, Lima, Peru; LGFYR.Lm13; 24 Feb. 2020 | OK095036 |
| *T. chocolata*; Chorrillos, Lima, Peru; LGFYR.Lm14; 24 Feb. 2020 | OK095037 |
| *T. chocolata*; Chorrillos, Lima, Peru; LGFYR.Lm15; 24 Feb. 2020 | OK095038 |
| *T. chocolata*; Chorrillos, Lima, Peru; LGFYR.Lm16; 24 Feb. 2020 | OK095039 |
| *T. chocolata*; Chorrillos, Lima, Peru; LGFYR.Lm17; 24 Feb. 2020 | OK095040 |
| *T. chocolata*; Chorrillos, Lima, Peru; LGFYR.Lm18; 24 Feb. 2020 | OK095041 |
| *T. chocolata*; Chorrillos, Lima, Peru; LGFYR.Lm19; 24 Feb. 2020 | OK095042 |
| *T. chocolata*; Chorrillos, Lima, Peru; LGFYR.Lm20; 24 Feb. 2020 | OK095043 |
| *T. chocolata*; Chorrillos, Lima, Peru; LGFYR.Lm21; 24 Feb. 2020 | OK095044 |
| *T. chocolata*; Chorrillos, Lima, Peru; LGFYR.Lm22; 24 Feb. 2020 | OK095045 |
| *T. chocolata*; Chorrillos, Lima, Peru; LGFYR.Lm23; 24 Feb. 2020 | OK095046 |
| *T. chocolata*; Chorrillos, Lima, Peru; LGFYR.Lm24; 24 Feb. 2020 | OK095047 |
| *T. chocolata*; Pisco, Ica, Peru; LGFYR.Ps1; 22 Sep. 2018 | OK095048 |
| *T. chocolata*; Pisco, Ica, Peru; LGFYR.Ps2; 22 Sep. 2018 | OK095049 |
| *T. chocolata*; Pisco, Ica, Peru; LGFYR.Ps3; 22 Sep. 2018 | OK095050 |
| *T. chocolata*; Pisco, Ica, Peru; LGFYR.Ps4; 22 Sep. 2018 | OK095051 |
| *T. chocolata*; Pisco, Ica, Peru; LGFYR.Ps5; 22 Sep. 2018 | OK095052 |
| *T. chocolata*; Pisco, Ica, Peru; LGFYR.Ps6; 22 Sep. 2018 | OK095053 |
| *T. chocolata*; Pisco, Ica, Peru; LGFYR.Ps7; 22 Sep. 2018 | OK095054 |
| *T. chocolata*; Pisco, Ica, Peru; LGFYR.Ps8; 22 Sep. 2018 | OK095055 |
| *T. chocolata*; Pisco, Ica, Peru; LGFYR.Ps9; 22 Sep. 2018 | OK095056 |
| *T. chocolata*; Pisco, Ica, Peru; LGFYR.Ps10; 22 Sep. 2018 | OK095057 |
| *T. chocolata*; Pisco, Ica, Peru; LGFYR.Ps11; 22 Sep. 2018 | OK095058 |
| *T. chocolata*; Pisco, Ica, Peru; LGFYR.Ps12; 22 Sep. 2018 | OK095059 |
| *T. chocolata*; Pisco, Ica, Peru; LGFYR.Ps13; 22 Sep. 2018 | OK095060 |
| *T. chocolata*; Pisco, Ica, Peru; LGFYR.Ps14; 22 Sep. 2018 | OK095061 |
| *T. chocolata*; Pisco, Ica, Peru; LGFYR.Ps15; 22 Sep. 2018 | OK095062 |
| *T. chocolata*; Pisco, Ica, Peru; LGFYR.Ps16; 22 Sep. 2018 | OK095063 |
| *T. chocolata*; Pisco, Ica, Peru; LGFYR.Ps17; 22 Sep. 2018 | OK095064 |
| *T. chocolata*; Pisco, Ica, Peru; LGFYR.Ps18; 22 Sep. 2018 | OK095065 |
| *T. chocolata*; Pisco, Ica, Peru; LGFYR.Ps19; 22 Sep. 2018 | OK095066 |
| *T. chocolata*; Pisco, Ica, Peru; LGFYR.Ps20; 22 Sep. 2018 | OK095067 |

**Table S2.** **Continue**

| **Species; locality; collectors name; collection code, date** | **GenBank ID** |
| --- | --- |
| *T. chocolata*; Pisco, Ica, Peru; LGFYR.Ps21; 22 Sep. 2018 | OK095068 |
| *T. chocolata*; Pisco, Ica, Peru; LGFYR.Ps22; 22 Sep. 2018 | OK095069 |
| *T. chocolata*; Marcona, Ica, Peru; LGFYR.Mc1; 19 Mar. 2019 | OK095070 |
| *T. chocolata*; Marcona, Ica, Peru; LGFYR.Mc2; 19 Mar. 2019 | OK095071 |
| *T. chocolata*; Marcona, Ica, Peru; LGFYR.Mc3; 19 Mar. 2019 | OK095072 |
| *T. chocolata*; Marcona, Ica, Peru; LGFYR.Mc4; 19 Mar. 2019 | OK095073 |
| *T. chocolata*; Marcona, Ica, Peru; LGFYR.Mc5; 19 Mar. 2019 | OK095074 |
| *T. chocolata*; Marcona, Ica, Peru; LGFYR.Mc6; 19 Mar. 2019 | OK095075 |
| *T. chocolata*; Marcona, Ica, Peru; LGFYR.Mc7; 19 Mar. 2019 | OK095076 |
| *T. chocolata*; Marcona, Ica, Peru; LGFYR.Mc8; 19 Mar. 2019 | OK095077 |
| *T. chocolata*; Marcona, Ica, Peru; LGFYR.Mc9; 19 Mar. 2019 | OK095078 |
| *T. chocolata*; Marcona, Ica, Peru; LGFYR.Mc10; 19 Mar. 2019 | OK095079 |
| *T. chocolata*; Marcona, Ica, Peru; LGFYR.Mc11; 19 Mar. 2019 | OK095080 |
| *T. chocolata*; Marcona, Ica, Peru; LGFYR.Mc12; 19 Mar. 2019 | OK095081 |
| *T. chocolata*; Marcona, Ica, Peru; LGFYR.Mc15; 19 Mar. 2019 | OK095082 |
| *T. chocolata*; Marcona, Ica, Peru; LGFYR.Mc17; 19 Mar. 2019 | OK095083 |
| *T. chocolata*; Marcona, Ica, Peru; LGFYR.Mc18; 19 Mar. 2019 | OK095084 |
| *T. chocolata*; Marcona, Ica, Peru; LGFYR.Mc19; 19 Mar. 2019 | OK095085 |
| *T. chocolata*; Marcona, Ica, Peru; LGFYR.Mc21; 19 Mar. 2019 | OK095086 |
| *T. chocolata*; Marcona, Ica, Peru; LGFYR.Mc22; 19 Mar. 2019 | OK095087 |
| *Stramonita biserialis;* Cañete, Lima, Peru; LGFYR.Sbi.317.5; 17 Apr. 2017 | OM333552 |
| *S. biserialis;* Santa, Ancash, Peru; LGFYR.Sbi.329.3; 30 Aug. 2017 | OM333553 |
| *S. biserialis;* Santa, Ancash, Peru; LGFYR.Sbi.329.7; 30 Aug. 2017 | OM333554 |
